# Supplementary material for: Pain education for patients with non-specific low back pain in Nepal: protocol of a feasibility randomised clinical trial (PEN-LBP Trial)
Source: BMJ Open. 2018 Aug 10;8(8):e022423. doi: 10.1136/bmjopen-2018-022423 (PMC6089285; doi:10.1136/bmjopen-2018-022423)
Supplement: Supplementary file 1 [file bmjopen-2018-022423supp001.pdf]

Explaining Pain in an hour

Curriculum for Nepalese with non-specific low back pain

Learner or patient characteristics

Learners are adult patients who present to a Physiotherapy Department in Kathmandu, Nepal for the management of their low back pain (LBP). This education will be delivered to patients with any duration of LBP. Every participant who understands and can speak Nepali can be the learner for the planned curriculum of Explain Pain. These individuals may have a very low literacy level. Patients who have diagnosed psychiatric illness will be excluded from this intervention.

Deliverer or physiotherapist

A physiotherapist (Saurab Sharma) who has undergone "Explain Pain" course twice (in 2015 and 2017) will deliver Explain Pain to all the participants in the study. The physiotherapist will be supervised by Prof. G. Lorimer Moseley, one of the developers of the concept.

Number of learners or patients

A total of 40 learners will participate in Explain Pain in this study. All interventions will be delivered individually to each participant. No other concurrent interventions will be provided to the patients with Explain Pain, however, patients will be encouraged to perform some exercises at home for one week.

Unique needs of the learners

These learners are from Nepal, many of them may not have any formal education, and education as the core component of treatment may be new to these patients. Thus, this group may be a challenging group to provide Explain Pain intervention. Therefore, the contents are simplified and adapted to their need to match their level of understanding, and culture.

Delivery methods

The study physiotherapist will deliver approximately an hour long session of one-on-one Explain Pain to every study participant. Every participant will be provided with a take away education booklet, which includes details of target concept, pictures and stories to strengthen their education provided by the physiotherapist face-to-face. Participants will read the booklet if they are educated, or the family members can read out for them if they cannot read themselves. All the participants will be provided with an additional audio-visual information on neurophysiology knowledge of pain, if they can operate these at home (or office) to reinforce the learning so that the participants hear this every day before the follow-up for assessment in a week.

Place

A room at a physiotherapy facility (Sahara Care Hospital) in Kathmandu, Nepal.

Patient consideration

1. Physiotherapist will extract learner's personal goal of treatment. Nepalese generally struggle to bring out their own goals in general, this will be a difficult task.
2. Involvement of family throughout the education session will be encouraged if they accompany the patients. Role of family members will be highlighted in the education program and prognosis of the individuals with LBP.
3. All the participants will be reminded to perform their home-based tasks (reading, listening/watching audio-visual support, and exercising) by a text message or a phone call every day for five days a week.

Aims

The deliverer intends to:

1. Advise patient that educate is an important aspect of treatment of pain.
2. Provide contemporary knowledge about pain biology in relation to low back pain in an individual face-to-face teaching and learning environment.
3. Provide a comprehensive patient education by providing relevant information on graded exposure, pacing, and self-management.
4. Use pictures, metaphors, and relevant stories related to pain to explain details and complexities related to pain.

Objectives

At the end of the session learner will:

1. Have contemporary knowledge of pain biology that is relevant to their low back pain.
2. Understand importance of pain knowledge as a therapy.
3. Use the pain knowledge in changing the danger messages into safety signals, and using exercises to pave the track for recovery.

Explain Pain Curriculum plan

Part 1: Question and answer **[10 – 15 minutes]**

Physiotherapist will first toss two questions.

Question 1- "*Is there anything in particular that you would like to learn about your low back pain, or pain in general?*"

Physiotherapist will answer to any questions that arise.

Question 2- *"Do you know what caused your low back pain? Can you please explain the cause of your low back pain from what you have understood, or what you have been told?"*

Address any misconceptions and acknowledge and appreciate healthy/sound understanding about their pain. Talk about scans if appropriate.

Part 2: Discuss the key concepts of pain biology. **[40 minutes]**

1. Pain is normal and almost everyone gets it in life.
2. Body sends danger signals, and brain decides whether to produce pain.
3. Learning about pain changes pain; and anything associated with it can influence it.
4. Body learns pain and becomes overprotective over time.
5. Additional concept: Pain and tissue damage are poorly related.

[see the table below for the details]

Part 3: Do you want to learn ways to train your system? **[5 – 10 minutes]**

Teach 1 – 2 ways to train the system.

End: Ask if the patient has a cell phone. If he or she has a cell phone, ask if he/she wants to receive a daily reminder to perform home-based tasks, and learn more about pain.

Record the number and send the information daily.

Explain Pain Curriculum Plan

SN	Target concepts	Time required	Other ways of expressing the target concept	Content	Delivery and resources	Reinforcement/ Experiential learning	Did the patient understand? Assessment
1	Pain is normal and almost everyone gets it in life.	10 minutes	• Everyone has some pain in lifetime so you are not alone.<br>• Pain is normal.<br>• Your LBP is yours, is unique to you and real, and only you can control your pain.	• There is no test for pain or love.<br>• Emotional and physical pain are one.<br>• Pain is always a conscious event.	• Stories<br>• Brief pain epidemiology	• Pain should not be a reason to worry about, and stop you from enjoying life, and fulfilling life goals.	• Ask- so who suffers pain or how many people suffer pain?<br><br>*Answer- almost everyone.*
2	Body sends danger signals, and brain decides whether to produce pain.	10 minutes	• How danger signal travels in the body and how pain is perceived.<br>• Brain is needed to create/ perceive pain.<br>• Human body has danger sensors not pain sensors.<br>• Pain depends on the balance between danger and safety.	• Nociceptive pathway.<br>• Pain is created in the brain.<br>• Ask patient if they had pain when they did not have tissue injury (or use aggravating factors).	• Use a picture/ animation of how danger signals reach brain?<br><br>• Use the earthquake story.	• Have you ever experienced having an injury and no pain?<br>• Have you ever experienced pain when there was no injury?<br>• Use the scale protectometer to describe.	• So what creates pain?<br><br>*Answer: brain!!!*
3	Learning about pain changes pain; and anything associated with it can influence it.	10 minutes	• Knowing about pain can reduce pain.<br>• Education is analgesic.<br>• Retraining your system can reduce sensitization.	• Understanding your pain can reduce your pain.<br>• Wrong understanding can increase your pain.	• Sikha's pain story.	• Hear other's pain stories and analyse how it can be changed.	

4	Body learns pain and becomes overprotective over time.	10 minutes	• Out of many outputs of the brain, pain is only one protective output.<br>• As pain persists, body systems can be over protective.<br>• Multiple systems protect us from threats, and allow us to learn and heal.<br>• You can train your body systems to be less protective.	• As pain persists, body becomes sensitized and over protective.<br>• This can be changed by training our systems.	• The bending and lifting story.	• Ask the patient what are the other symptoms they get with pain? [Examples are: sweating, no sleep, stress, anxiety, fear, anger etc].	• Ask, do you understand this and think if this is logical?<br>• Reinforce this by saying, this is also scientific.
Optional	Pain and tissue damage are poorly related	5 minutes	• Pain is an unreliable indicator of tissue damage.<br>• Pain and scans do not correlate.	• Tissue stop hurting a long time before they heal.<br>• Recent evidences regarding poor correlation between pain and scan reports.	Bad scans in pain free people.	Reinforce by saying degeneration is like greying of hair, which is normal ageing process. It does not hurt.	
