# Supplementary material for: Pain education for patients with non-specific low back pain in Nepal: protocol of a feasibility randomised clinical trial (PEN-LBP Trial)
Source: BMJ Open. 2018 Aug 10;8(8):e022423. doi: 10.1136/bmjopen-2018-022423 (PMC6089285; doi:10.1136/bmjopen-2018-022423)
Supplement: Author's manuscript [file bmjopen-2018-022423.draft_revisions.pdf]

BMJ Open is committed to open peer review. As part of this commitment we make the peer review history of every article we publish publicly available.

When an article is published we post the peer reviewers' comments and the authors' responses online. We also post the versions of the paper that were used during peer review. These are the versions that the peer review comments apply to.

The versions of the paper that follow are the versions that were submitted during the peer review process. They are not the versions of record or the final published versions. They should not be cited or distributed as the published version of this manuscript.

BMJ Open is an open access journal and the full, final, typeset and author-corrected version of record of the manuscript is available on our site with no access controls, subscription charges or pay-per-view fees (http://bmjopen.bmj.com).

If you have any questions on BMJ Open's open peer review process please email
info.bmjopen@bmj.com

BMJ Open

Pain Education for patients with non-specific low back pain in Nepal: Protocol of a feasibility randomized clinical trial (PEN-LBP Trial).

Journal:	*BMJ Open*
Manuscript ID	bmjopen-2018-022423
Article Type:	Protocol
Date Submitted by the Author:	16-Feb-2018
Complete List of Authors:	Sharma, Saurab; Kathmandu University School of Medical Sciences, Department of Physiotherapy; University of Otago Dunedin School of Medicine, Orthopedic Surgery Section, Department of Surgical Sciences<br>Jensen, Mark; University of Washington<br>Moseley, G.; Sansom Institute for Health Research, University of South Australia<br>Abbott, J. Haxby; University of Otago, Department of Surgical Sciences, Dunedin School of Medicine
Keywords:	Explain pain, Explaining Pain, Back pain < ORTHOPAEDIC & TRAUMA SURGERY, Physiotherapy, Low-income country, Developing country

SCHOLARONE™
Manuscripts

For peer review only

Title: **P**ain **E**ducation for patients with non-specific low back pain in **N**epal: Protocol of a feasibility randomized clinical trial (PEN-LBP Trial).

Authors: Saurab Sharma [1, 2]

Mark P. Jensen [3]

Lorimer G. Moseley [4]

J. Haxby Abbott [2]

Affiliations: [1] Department of Physiotherapy, Kathmandu University School of Medical Sciences, Dhulikhel, Nepal

[2] Centre for Musculoskeletal Outcomes Research, Dunedin School of Medicine, University of Otago, Dunedin, New Zealand

[3] Department of Rehabilitation Medicine, University of Washington, Seattle, USA

[4] School of Health Sciences, University of South Australia, Australia.

Present address of corresponding author:
Saurab Sharma
Assistant Professor, Department of Physiotherapy
Kathmandu University School of Medical Sciences
Dhulikhel Hospital Kathmandu University Hospital
Dhulikhel, P.O. Box No.: 11008, Nepal
Office fax: +977 1 490 707
saurabsharma1@gmail.com
Phone: +977 984 163 4043

For peer review only - http://bmjopen.bmj.com/site/about/guidelines.xhtml

ABSTRACT

Introduction: Low back pain (LBP) is the leading cause of years lived with disability in Nepal and elsewhere. Management of LBP that is evidence-based, easily accessible, cost-effective, and culturally appropriate is desirable. The primary aim of this feasibility randomized clinical trial is to determine if it is feasible to conduct a full randomized clinical trial evaluating the effectiveness of pain education as an intervention for patients in Nepal with LBP, relative to guideline-based usual care. The findings of the study will inform the planning of a full clinical trial, and if any modifications are required to the protocol before undertaking a full trial.

Methods/analysis: This protocol describes an assessor-blinded feasibility clinical trial investigating feasibility of pain education intervention in patients with non-specific low back pain in a rehabilitation hospital in Kathmandu, Nepal. Forty patients with LBP will be randomly allocated to either pain education or guideline-based usual care (control). Outcomes will be assessed at baseline and at a one-week post-treatment. The primary outcomes are related to feasibility, including: (1) participant willingness to participate in a randomized clinical trial, (2) feasibility of assessor blinding, (3) eligibility and recruitment rates, (4) acceptability of screening procedures and random allocation, (5) possible contamination between the groups, (6) intervention credibility, (7) intervention adherence , (8) treatment satisfaction, and (9) difficulty in understanding the interventions being provided.

For peer review only - http://bmjopen.bmj.com/site/about/guidelines.xhtml

Ethics/dissemination: The protocol was approved by Nepal Health Research Council (NHRC; registration number: 422/2017) and University of Otago Human Ethics Committee for Health (registration number: H17/157). Any change to study protocol will be informed to both the IRBs. The results of the study will be presented at national and international conferences, and published in a peer-reviewed journal.

Trial registration number: NCT03387228.

Keywords: Explain pain; Explaining pain; Back pain; Physiotherapy; Low-income country; Developing country

For peer review only

Strengths and limitations of this study

- This protocol describes the development of a culturally-adapted pain education intervention in Nepal.
- This study will describe whether or not a full trial based on the pilot intervention will be feasible.
- Assessor will be blinded to group allocation of the participants.
- Therapist and patient blinding is not possible in this study.
- Assessment of the effectiveness of the intervention is not the primary aim of this study.

For peer review only - http://bmjopen.bmj.com/site/about/guidelines.xhtml

INTRODUCTION

Low back pain (LBP) is a highly prevalent health condition worldwide.[1] It is the leading cause of disability,[2] and imposes huge economic burden to the society in both developed and developing countries.[3-6] LBP (with neck pain) is the most common health condition contributing to years lived with disability in Nepal.[2] Although the prevalence of LBP is high in Nepal, ethnographic research has noted that LBP-related disability may be low in rural areas,[7] perhaps due to the very low socioeconomic status of individuals living in rural Nepal, which forces them to keep working despite the presence of pain. Consistent with this idea, another study highlighted that 80% of people with chronic pain in Nepal continue to work.[6] However, it is alarming that number of spine surgeries for spinal pain has been increasing in Nepal over the years,[8] despite lack of evidence supporting efficacy for this treatment.[1 9]

Interventions for management of LBP

Many interventions have been investigated for the management of LBP. These include surgery,[10 11] pharmacotherapy,[12-17] exercises,[18-20] advice for self-management including advice to remain physically active,[11] and psychological therapies.[21 22] As alluded to earlier, biomedically-focused interventions such as surgery and pharmacotherapy are not recommended for a non-specific LBP as the evidence does not support their effectiveness.[1 14] Moreover, they are associated with significant risks for adverse events and are costly.[5]

For peer review only - http://bmjopen.bmj.com/site/about/guidelines.xhtml

Clinical practice guidelines recommend exercise, self-management, and education for LBP,[23] with the evidence indicating that exercise has the largest beneficial effects in the prevention[24] and the management[18] of LBP. The most common problem with exercise treatment for LBP is treatment adherence.[25] Patient education, on the other hand, is a cost-effective therapy,[26] which has no known negative side effects, as long as it is based on accurate neurophysiological knowledge.

Pain education for LBP

Patient education for LBP that has been investigated in RCTs are basically of two types: biomedical education and pain biology education.[27] The first refers to educating patients about vertebral anatomy. However, biomedical education has shown to be ineffective and may even cause harm.[27] On the other hand, the second type of education – pain biology education (hereafter called as "pain education") has been shown to have positive effects on both pain and disability.[28 29] Pain education is structured education program with specific aims and objectives.[30] This intervention has a list of target key concepts to be delivered and includes the curriculum contents to deliver the key concepts using up-to-date pain science knowledge, stories, and metaphors.

It has been previously hypothesized that this type of education program utilizing metaphors and stories may be an effective intervention in Nepalese with chronic pain.[31] However, the pain education materials that have been developed in western cultures should not be assumed will be valid and equally effective in reducing pain and disability in a non-western culture. When delivering, to a non-western culture, pain education that was developed in western culture, (significant) cultural adaptations of the education

For peer review only - http://bmjopen.bmj.com/site/about/guidelines.xhtml

materials may be required to make it suitable for the target population, as culturally inappropriate education or information may not produce desirable results.

Therefore, in order to evaluate the effectiveness of pain education in individuals with non-specific low back pain from Nepal, culturally-appropriate pain education materials should first be developed for Nepal, specifically. However, it is possible that the adaptations made could potentially reduce its effectiveness. Thus, before testing the adapted pain education in a full clinical trial, a feasibility study is needed in order to determine if a full clinical trial based on the adapted intervention is warranted, or if additional modifications may be needed prior to performing the full trial.

Why the feasibility trial?

We propose a feasibility trial because: (1) the intervention (i.e., pain education) will need significant cultural adaptation, although it has been evaluated for efficacy previously in other languages and western cultures; (2) the adapted intervention has never been investigated for its efficacy or effectiveness before; (3) the population in question (individuals with extremely low socioeconomic status and educational attainment in Nepal) is unique; and (4) a high quality clinical trial in individuals with LBP has not been conducted in Nepal, to our knowledge, and we therefore do not know if a full trial is feasible. The findings from the proposed feasibility study will inform the planning and design of a full trial, if the results indicate that a full trial is warranted.

The results of the full trial will have a significant clinical implication for the management of LBP in Nepal, and similar cultures, providing empirical evidence pain education is a viable treatment option for the management of LBP, and if it is effective in reducing

For peer review only - http://bmjopen.bmj.com/site/about/guidelines.xhtml

pain, disability, and emotional distress. This will reduce the need of medications, surgeries, and health care utilization eventually minimizing the cost of treatment for LBP and reducing economic burden of the low-income country where health-care is expensive.

Aims and objectives

The primary aim of this feasibility randomized study is to evaluate the feasibility of a full randomized clinical trial (as designed in this protocol) for assessing the effects of pain education as an intervention for patients with LBP in a physiotherapy facility in Nepal after developing culturally-appropriate, evidence-based pain education materials. The primary objectives of the study are related to feasibility of randomized clinical trial, specifically: (1) willingness to participate in a randomized controlled trial, (2) feasibility of assessor blinding, (3) eligibility and recruitment rates, (4) acceptability of screening procedures and random allocation, (5) possible contamination between the groups, (6) intervention credibility for patients with LBP, (7) intervention adherence, (8) treatment satisfaction, and (9) difficulty in understanding the intervention being provided.

METHODS AND ANALYSIS

Study Design and setting

This is a feasibility study that is being performed to determine if a full randomized controlled trial (RCT) can be successfully conducted using the procedures and protocol of the feasibility study, or if modifications of the feasibility study protocol are needed

For peer review only - http://bmjopen.bmj.com/site/about/guidelines.xhtml

prior to conducting the full trial. The study findings will inform the design of the full trial, if a full trial is found to be feasible.[32]

The definition of a feasibility study highlights the question, *"Can this study be done?"* The SPIRIT statement,[33] the CONSORT statement extension to pilot and feasibility randomized trial,[32] was followed in the planning of the study and reporting of the protocol.

The study will be an assessor-blinded, two-arm, feasibility randomized clinical trial (RCT). Ethical approval has been obtained from the Nepal Health Research Council (NHRC, reference number: 422/2017) and University of Otago Human Ethics Committee (reference number: H17/157). The study is registered in clinicaltrials.gov (trial registration number: NCT03387228). The study will be conducted in the Sahara Rehabilitation Hospital, Kathmandu, Nepal which is a private hospital in Kathmandu, Nepal.

Overview of the study

Advertisement of the trial will be made in social media and all the patients with low back pain presenting at study site will be invited to participate. Interested candidates will be screened for eligibility. Eligible patients with non-specific LBP will then be enrolled in the trial and be randomly assigned to one of the two study groups. All the participants in the experimental group will receive pain education and those in the control group will receive evidence based usual physiotherapy care. All the participants will be assessed at baseline, and one week following treatment. Details describing the schedule of

For peer review only - http://bmjopen.bmj.com/site/about/guidelines.xhtml

enrolment, interventions, and assessment are presented in Table 1, in the manner recommended by SPIRIT checklist.[33]

[Insert table 1 about here]

Participants

Patients with non-specific low back pain seeking rehabilitative services at Sahara Care Hospital will be invited to participate in the feasibility clinical trial. Interested participants will be screened by a research assistant (physiotherapist by training) involved in the research.

Inclusion Criteria

Nonspecific LBP (LBP other than those excluded, see exclusion criteria below) of any duration with pain primarily localized between T12 and gluteal folds, in patients aged 18 years or older, with average pain intensity reported as moderate, severe, or very severe on a PROMIS five-point PROMIS Pain Intensity Short-form Scale[34] over the past week, and who is a Nepalese and is able to understand and speak Nepali fluently will be included in the study.

Exclusion Criteria

Participants with likely specific causes of LBP will be excluded using a triage procedure as suggested by Bardin and colleagues.[35] This includes exclusion of participants having history of prolonged use of corticosteroid, history of malignancy, recent history of fever or chills, history of other diseases associated with compromise in immune system,

For peer review only - http://bmjopen.bmj.com/site/about/guidelines.xhtml

history of recent spinal surgery or dental procedures, recent history of trauma to spine or a fracture of a spine, history of bladder and bowel dysfunction, and history of perineal or saddle anaesthesia. Additionally, current pregnancy, and history of diagnosed mental health conditions that would limit adherence to the trial procedures will be excluded.

Sample Size

For a feasibility study, it is inappropriate to calculate sample size based on desired statistical power to detect a treatment effect,[36] because, the primary aim of the study is to assess if a full trial can or should be conducted. Feasibility outcomes are descriptive in nature; therefore inferential statistics regarding treatment effects will not be computed. To achieve the primary objectives related to feasibility outcomes, the research team estimated that 40 participants would be adequate.[37] Twenty patients will be randomly allocated to each treatment condition.

Participant screening and recruitment

Consecutive participants with non-specific LBP will be invited to participate in this study. The study purpose and procedures will be described to potential participants. This will include information about the benefits and potential harms of the intervention, the time required for the completion of the study, follow-up duration, voluntariness of participation, cost of participation, and the rights to withdraw from the study at any point. A study information sheet will be provided to all potential participants.

For peer review only - http://bmjopen.bmj.com/site/about/guidelines.xhtml

If the potential participants are interested in participating, they will be screened for eligibility by a research assistant who is a physiotherapist. If the participants are found eligible, informed consent will be obtained. For those who cannot sign the consent, a witness will sign on their behalf, or the study participant will provide a thumb print on the form for those who cannot write or sign the form as per the ethical guidelines provided by NHRC. We will include uneducated patients who cannot sign an informed consent in order to increase the inclusion of uneducated or low education group, given that 31% people in Nepal who are five years old or more cannot read and write.[38] Additionally, exploration of feasibility of pain education in those with no schooling or low educational attainment is important in order to inform clinical practice.

Participants will be informed that they will receive one of the two treatments randomly. It will be highlighted that both of the treatment options are thought to be effective for LBP, and that the goal of the main study is to compare the interventions, however the current study will more specifically evaluate the feasibility of such a study.

Group Allocation, Randomization and Blinding

Random number sequence, in random blocks of 4 and 6, will be generated using www.randomization.com, by a researcher (JHA) who is not involved in recruitment process. Allocation concealment will be performed using opaque, sealed envelopes. The participants will be allocated to one of the two groups by a hospital staff-member who is not the assessor. The two groups will be: (1) Group 1: Pain education group (PEG), and (2) Group 2: Control group (CG).

For peer review only - http://bmjopen.bmj.com/site/about/guidelines.xhtml

Intervention

The TIDieR (Template for Intervention Description and Replication) Checklist was followed when planning the study intervention.[39 40] Manuals of standard operating procedures (MSOPs) will be followed during the delivery of the interventions in both the groups. This will ensure treatment uniformity and fidelity. It is not possible to blind the intervention providers based on the design of the study.

Participants in intervention group (PEG) will receive detailed pain education as described in the next paragraph. Participants in control group (CG) will receive usual care during the study duration. After the completion of the follow-up assessment, study participants in both the groups will receive usual physiotherapy treatment being provided by physiotherapists at Sahara Care Hospital. Participants in both the groups will be encouraged not to seek for other medical care for LBP during the 1-week study period, unless they have to. If they do undertake other forms of treatment, they will be requested to report this during the follow-up assessment, and this will be recorded.

Pain education group (PEG)

Pain education will be delivered to the PEG only. A number of investigators have described pain education as a treatment option for individuals with LBP. We will use the pain education handouts in Nepali for Nepalese with pain based on the resources developed by Moseley and Butler– called Explain Pain.[30 41 42] It has evolved in over 15 years[43] and undergone changes and advances.[30 41 42]

The use of pain education in the management of patients with chronic LBP has been supported by research.[44] A recent systematic review showed moderate to high quality

For peer review only - http://bmjopen.bmj.com/site/about/guidelines.xhtml

evidence that the primary care based patient education positively influences patient reassurance compared to no education or control education in both short and long term.[45] Pain education is delivered to provide reassurance, which means removal of fear and concerns about illness.[46] Reassurance is among the core aspects of management of patients with non-specific problems such as LBP.[47] Although there are no gold standard ways to provide reassurance and alleviate fear and concerns about a disease or its consequences, empathy and collaboration are thought to play important role.[48] Intervention will be provided by the lead investigator (SS) who is trained in the delivery of pain education, and has around 10 years of experience in the management of musculoskeletal disorders, including LBP.

In order to develop the pain education resources in Nepali, the first step is the development of curriculum for pain education for patients with LBP[30] in Nepal. The curriculum was first outlined in English (by SS) in the similar manner recommended by the developers,[30] and was reviewed and approved by both the developers (see supplementary material). Based on the curriculum, pain education materials and patient handouts were created (by SS) in Nepali. The clinical cases and patient stories that were compiled are actual stories collected from patients and clinicians in Nepal, but will be anonymously shared to provide reasoning based on contemporary pain biology knowledge.[30] Pictures that are found in Explain Pain resource materials[30 41 42] were adapted in the Nepali version, and newer culturally appropriate pictures were created. The pain education handout and materials that are produced in Nepali was proof-read and pre-tested in five Nepalese with LBP, and corrected, before we use it in the feasibility study participants. The final handouts were then printed for the use in the

For peer review only - http://bmjopen.bmj.com/site/about/guidelines.xhtml

current study. This adapted process will ensure that the Nepali pain education materials produced are valid and culturally appropriate. However, for the purpose of the full trial, the difficulty in understanding the treatment will be assessed in the current feasibility study, and any modifications required will be made.

Dosage of the PEG intervention

A single approximately one hour pain education will be delivered to the PEG, because evidence indicates that: (1) interventions as brief as five minutes have been shown to have reassuring effects lasting for up to a year,[45] and a single consultation have been found to be as reassuring as the multiple session interventions.[48] We prefer a single session treatment over multiple session treatment, because for many patients in rural Nepal, it is difficult to deliver a multiple session treatment in reality. Thus, we plan to provide a single session delivering key concepts of Explain Pain, which will be reinforced by providing patient handouts to look at and read at home. In fact, a one hour session should be adequate to cover the key 'Explain Pain' concepts by keeping the content simple and jargon-free. Use of a plain language in the health-related education is important to adapt in low health literacy.[49]

Home advice for PEG group

A printed handout of the pain education material will be provided only to the study participants in the PEG. Participants will be advised to read them, followed by perform physical activity including walking for approximately 30 minutes. Education accompanied by written information has reported to yield the largest effects on fear-

For peer review only - http://bmjopen.bmj.com/site/about/guidelines.xhtml

component of emotional distress.[26] However, education level of Nepalese is low (65%), we will adapt the written materials to incorporate many more images than text.[38]

For those who cannot read, the family member(s) will be encouraged to read out the materials to them. To complement the written materials, we will also provide an audio recording of the pain education session to the patients as an online URL link to Nepali patient education material stored in the cloud, or will be copied to their smart phones or both for those who have the facilities to use them. The pain education advice will be directed towards reducing brain's perception movement and exercise as a threat encouraging participants to slowly pace the movement, physical activity, and exercise. This is thought to desensitize the sensitive nervous system, and improve function. A written instruction to perform general exercises will be sent to the participants. A reminder to perform home exercises will be sent to all participants for a total of five days in the week.

Control group (CG)

The intervention will be provided by the physiotherapists working at the study site. Treatment integrity in the control group will be improved by providing an interactive seminar to all the physiotherapists delivering control group intervention by the lead researcher. The seminar will incorporate evidence-based information, including assessment and management of LBP based on the current recommendations from clinical practice guidelines.[50-53] Research articles, and simplified evidence-based summary will be provided to the physiotherapists before the interactive seminar for self-study. At the end of the seminar, a brief multiple-choice quiz will be conducted for the

For peer review only - http://bmjopen.bmj.com/site/about/guidelines.xhtml

study physiotherapists in order to verify the understanding of the evidence-based knowledge and management of LBP of the therapists.

Intervention in the control group

The control participants will receive physiotherapy care based on the recent clinical practice guidelines from three different western countries- (1) American College of Physicians, USA (2017), (2) National Institute for Health and Care Excellence, UK (2016), and (3) Toward Optimized Practice, Canada (2015).[50-53] These guidelines are used because they are recent and highly regarded. We did not find any evidence based clinical practice guidelines for the management of LBP in Nepal, or other developing countries. The control group interventions were derived by comparing the recommendations made by each of the three guidelines on specific management strategies. The management for our study were selected, (1) if two or more guidelines recommended an intervention, (2) if all the authors of the current study agreed in the intervention, (3) if the interventions were culturally accepted and feasible to deliver in Nepal, (4) the intervention were considered appropriate to deliver to the patients at first contact, and (5) total duration of all the interventions would sum up to one hour (approximately) to make the contact hour comparable to approximate one hour of pain education session in the experimental group. Similarities and difference in the clinical practice guidelines across the countries are common, and are influenced highly from the experience of the expert committee responsible for clinical practice guideline, and local practice trends.[54] For this reason, we chose to adapt the guideline to fit the expertise of

For peer review only - http://bmjopen.bmj.com/site/about/guidelines.xhtml

the local physiotherapist, practice trends that are widely accepted, and feasibility of delivering within the context of the study.

This control group intervention will comprise of: (1) education (advice to remain active, education about prognosis of low back pain for acute LBP, avoid bed rest and braces)[51 53 55] for 10-15 minutes, including time spent to listen to their pain story; (2) massage[53 55] to back for about 10 minutes; (3) superficial heat[51 53] for 10-15 minutes; and (4) static cycling or (treadmill) walking with the aim to promote physical activity[51 53 55] including any rest period for a total of approximately 20 minutes. Although superficial heat is recommended in the acute/subacute LBP,[51 53] we included this as a common treatment for all types of LBP including chronic LBP, which could be a part of self-management. Any forms of electrotherapy and acupuncture will not be offered to the study participants.[50] The intervention will strictly exclude the use of pain biology education.

Dosage of intervention for the CG

The intervention will last for a total of one hour to match with the experimental group.

Home advice for the CG

Participants will be advised to self-manage their back pain based on the information provided. Home exercise leaflet with emphasis on the value of exercise to increase strength and endurance, followed by a 30 minutes walking. Advice preceding the exercises will state that exercises are needed to keep you strong, healthy, and pain-free. A reminder to perform home exercises will be sent to all the participants for five days during the week.

For peer review only - http://bmjopen.bmj.com/site/about/guidelines.xhtml

Measures

The details of primary feasibility outcomes are presented in table 2.

[Insert table 2 about here]

All but one secondary outcome measures have been shown to be reliable and valid in Nepali populations. The single item measure on quality of life is not yet validated at the time of writing the protocol, however, we have included it as a secondary outcome measure because it is a recommended measure by core-outcome sets in clinical trials for low back pain.[56] The hypothesize that this measure is comprehensible and will show adequate validity in Nepalese sample. We will evaluate the validity of the measure before using it in the full clinical trial.

A research assistant will interview all the study participants via interview to make the study procedures consistent, and to include all the participants with no education and low education. They will be administered by a registered physiotherapist, who will be blind to group assignment. The principal investigator will train the research assistant for consistent and quality data collection. The research assistant will make a phone call to remind the participant for the post-treatment assessment after one week. Secondary measures include the four Patient-Reported Outcome Measurement Information System (PROMIS) short-form measures assessing Pain Interference, Pain Intensity, Sleep Disturbance, and Depression, as well as the 13-item Pain Catastrophizing Scale (PCS), Connor-Davidson Resilience Scale (CD-RISC), Global Rating of Change (GROC), and a single-item quality of life (QOL) scale. All the items in each PROMIS measure will be summed to obtain raw scores for each scale. The raw scores of each measure will then

For peer review only - http://bmjopen.bmj.com/site/about/guidelines.xhtml

be converted to T-scores, with a mean of 50 and SD of 10 and recorded (www.assessmentcenter.net). Details of the measures with their measurement properties are presented in table 3.

[Insert table 3 about here]

The risk of adverse events in both the groups are very low. Home exercises such as walking and back-specific exercises are emphasized on choosing a level that is comfortable for the patient. Duration, and pace of exercises should be quickly corrected if the patient feels the initial level is too high.[57] Participants will be asked to record any adverse events if occurred, and report to the researcher. Adverse events in both the groups will be reported and compared between the groups.

Additional measures

Additional questionnaires will be administered to obtain data related to (1) socio-demographic information (age, sex, education level, employment status, income, religion and ethnicity); (2) pain history including duration of pain, aggravating and relieving factors, other associated comorbidities; and (3) pain location using drawings. Other information such as resources required to conduct the trial (e.g., cost), time required to complete the recruitment of desired number of participants will also be recorded.[58] Total duration of home exercises in each group will also be recorded.

Criteria for feasibility

For peer review only - http://bmjopen.bmj.com/site/about/guidelines.xhtml

The results of this feasibility trial will indicate if the study as designed is feasible which will inform the decision of progressing to a full trial with the recommendations. The decision will be one of the following: (1) do not proceed to a full trial if any pre-planned changes may not help improve the feasibility; (2) modify the protocol further prior to conducting a full trial; (3) continue with the full trial using the same procedures used in the feasibility trial without modifications, however, monitor the study procedures closely; and (4) continue without modifications, as it is in the feasibility trial, close monitoring is not required.[59] The criteria for the feasibility are presented in Table 4.

[Insert Table 4 about here]

Limitation of the Study

As this is a feasibility study, the results of the current study will not provide findings regarding the efficacy of the interventions being tested. The study will only evaluate if this research study is viable as a full trial, and inform any recommendations for modification of the protocols for the full trial resulting from the findings of this feasibility trial.

Plan for Supervision and Monitoring

The study will be conducted, and monitored by the lead investigator under the supervision of the co-authors (JHA, MPJ), with assistance from research assistant(s). All the ethical principles as provided by Declaration of Helsinki will be followed by all the members of this research throughout the study. The investigators will not violate any of the rules and ethical principles of Nepal Health Research Council (NHRC). Internal data

For peer review only - http://bmjopen.bmj.com/site/about/guidelines.xhtml

monitoring team will monitor the trial. Monitoring for the NHRC will be regulated by the primary investigator and followed by all researchers and research assistants involved in the study.

Plan for data integrity and management

The research data will be collected by a research assistant who will be trained to collect the research data, manage the data by compiling in a file for individual patient. Participant identifiers (including name, address, and contact information) will be removed from the research data, and will be stored separately. Data will be entered in Microsoft Excel. Identification of the groups as intervention and control group will be removed from the excel sheet. Research data will be monitored weekly by scrutinizing entered data. Any errors in entry will be identified (if any) and amended. Consent forms will be scanned and stored in password protected computers of the lead researcher and at the University of Otago along with other research data files.

Data analysis plan

Descriptive statistics will be computed to describe the baseline and demographic characteristics of the study participants. As it is a feasibility study, level of significance and hypothesis testing regarding treatment efficacy will not be performed. Effect sizes representing between-group differences in change in the primary and secondary outcomes will be computed, but these effect sizes will not be considered as a criteria for sample size estimation for the full trial, nor as a criteria to proceed to the full trial, because of the inadequate power of the current feasibility study. Treatment effects for

For peer review only - http://bmjopen.bmj.com/site/about/guidelines.xhtml

the secondary outcome measures will be presented as means, standard deviations, and confidence intervals of the means. Difference between the mean scores of each secondary outcome will be compared with the minimum important change (MIC) values of the outcome measures, if the MIC scores are available. The analyses plan of the primary feasibility objectives are described in table 2.

Authors' contribution

SS, JHA, and MPJ conceived the idea of the trial. Trial was designed by SS, JHA, MPJ, and GLM. SS prepared the first draft of the protocol. Successive drafts were contributed by JHA, MPJ, and GLM. The final version of the protocol was approved by all the authors.

Funding

None

Competing interests

GLM is the developer of the experimental intervention, however, he will not have any influence in the analysis of data. Other authors have no conflict of interest.

Ethics approval

The protocol was approved by Nepal Health Research Council (ref number 422/2017) and University of Otago Human Research Committee (Health) (ref number H17/157).

For peer review only - http://bmjopen.bmj.com/site/about/guidelines.xhtml

Acknowledgement

The authors would like to thank David Butler and Tim Cocks from Neuro-Orthopedic Institute (NOI) in Adelaide for their input during the development of curriculum for explaining pain for this study, and Adrian Traeger for comments during planning of the research protocol.

For peer review only

For peer review only - http://bmjopen.bmj.com/site/about/guidelines.xhtml

References:

1. Balague F, Mannion AF, Pellise F, et al. Non-specific low back pain. *Lancet* 2012;379(9814):482-91. doi: 10.1016/S0140-6736(11)60610-7

2. Global Burden of Disease Study C. Global, regional, and national incidence, prevalence, and years lived with disability for 301 acute and chronic diseases and injuries in 188 countries, 1990-2013: a systematic analysis for the Global Burden of Disease Study 2013. *Lancet* 2015;386(9995):743-800. doi: 10.1016/S0140-6736(15)60692-4

3. Walker BF, Muller R, Grant WD. Low back pain in Australian adults: the economic burden. *Asia Pac J Public Health* 2003;15(2):79-87. doi: 10.1177/101053950301500202

4. Hong J, Reed C, Novick D, et al. Costs associated with treatment of chronic low back pain: an analysis of the UK General Practice Research Database. *Spine (Phila Pa 1976)* 2013;38(1):75-82. doi: 10.1097/BRS.0b013e318276450f

5. Machado GC, Maher CG, Ferreira PH, et al. Trends, Complications, and Costs for Hospital Admission and Surgery for Lumbar Spinal Stenosis. *Spine (Phila Pa 1976)* 2017 doi: 10.1097/BRS.0000000000002207

6. Bhattarai B, Pokhrel PK, Tripathi M, et al. Chronic pain and cost: an epidemiological study in the communities of Sunsari district of Nepal. *Nepal Med Coll J* 2007;9(1):6-11. [published Online First: 2007/06/28]

7. Anderson RT. An orthopedic ethnography in rural Nepal. *Med Anthropol* 1984;8(1):46-59. doi: 10.1080/01459740.1984.9965888

For peer review only - http://bmjopen.bmj.com/site/about/guidelines.xhtml

8. Bajracharya S, Singh M, Singh GK, et al. Clinico-epidemiological study of spinal injuries in a predominantly rural population of eastern Nepal: A 10 years' analysis. *Indian J Orthop* 2007;41(4):286-9. doi: 10.4103/0019-5413.36988

9. Deyo RA, Nachemson A, Mirza SK. Spinal-fusion surgery - the case for restraint. *N Engl J Med* 2004;350(7):722-6. doi: 10.1056/NEJMsb031771

10. Fritsch CG, Ferreira ML, Maher CG, et al. The clinical course of pain and disability following surgery for spinal stenosis: a systematic review and meta-analysis of cohort studies. *Eur Spine J* 2017;26(2):324-35. doi: 10.1007/s00586-016-4668-0

11. Fernandez M, Ferreira ML, Refshauge KM, et al. Surgery or physical activity in the management of sciatica: a systematic review and meta-analysis. *Eur Spine J* 2016;25(11):3495-512. doi: 10.1007/s00586-015-4148-y

12. Machado GC, Maher CG, Ferreira PH, et al. Non-steroidal anti-inflammatory drugs for spinal pain: a systematic review and meta-analysis. *Ann Rheum Dis* 2017;76(7):1269-78. doi: 10.1136/annrheumdis-2016-210597

13. Enthoven WTM, Roelofs PD, Koes BW. NSAIDs for Chronic Low Back Pain. *JAMA* 2017;317(22):2327-28. doi: 10.1001/jama.2017.4571

14. Saragiotto BT, Machado GC, Ferreira ML, et al. Paracetamol for low back pain. *Cochrane Database Syst Rev* 2016(6):CD012230. doi: 10.1002/14651858.CD012230

15. Mathieson S, Maher CG, McLachlan AJ, et al. Trial of Pregabalin for Acute and Chronic Sciatica. *N Engl J Med* 2017;376(12):1111-20. doi: 10.1056/NEJMoa1614292

For peer review only

For peer review only - http://bmjopen.bmj.com/site/about/guidelines.xhtml

16. Abdel Shaheed C, Maher CG, Williams KA, et al. Efficacy and tolerability of muscle relaxants for low back pain: Systematic review and meta-analysis. *Eur J Pain* 2017;21(2):228-37. doi: 10.1002/ejp.907

17. Abdel Shaheed C, Maher CG, Williams KA, et al. Efficacy, Tolerability, and Dose-Dependent Effects of Opioid Analgesics for Low Back Pain: A Systematic Review and Meta-analysis. *JAMA Intern Med* 2016;176(7):958-68. doi: 10.1001/jamainternmed.2016.1251

18. Saragiotto BT, Maher CG, Yamato TP, et al. Motor Control Exercise for Nonspecific Low Back Pain: A Cochrane Review. *Spine (Phila Pa 1976)* 2016;41(16):1284-95. doi: 10.1097/BRS.0000000000001645

19. Macedo LG, Saragiotto BT, Yamato TP, et al. Motor control exercise for acute non-specific low back pain. *Cochrane Database Syst Rev* 2016;2:CD012085. doi: 10.1002/14651858.CD012085

20. Saragiotto BT, Maher CG, Yamato TP, et al. Motor control exercise for chronic non-specific low-back pain. *Cochrane Database Syst Rev* 2016(1):CD012004. doi: 10.1002/14651858.CD012004

21. Cherkin DC, Anderson ML, Sherman KJ, et al. Two-Year Follow-up of a Randomized Clinical Trial of Mindfulness-Based Stress Reduction vs Cognitive Behavioral Therapy or Usual Care for Chronic Low Back Pain. *JAMA* 2017;317(6):642-44. doi: 10.1001/jama.2016.17814

22. Gatchel RJ, Rollings KH. Evidence-informed management of chronic low back pain with cognitive behavioral therapy. *Spine J* 2008;8(1):40-4. doi: 10.1016/j.spinee.2007.10.007 [published Online First: 2008/01/01]

For peer review only - http://bmjopen.bmj.com/site/about/guidelines.xhtml

23. Delitto A, George SZ, Van Dillen LR, et al. Low back pain. *J Orthop Sports Phys Ther* 2012;42(4):A1-57. doi: 10.2519/jospt.2012.0301 [published Online First: 2012/04/03]

24. Steffens D, Maher CG, Pereira LS, et al. Prevention of Low Back Pain: A Systematic Review and Meta-analysis. *JAMA Intern Med* 2016;176(2):199-208. doi: 10.1001/jamainternmed.2015.7431

25. Maher C, Underwood M, Buchbinder R. Non-specific low back pain. *Lancet* 2017;389(10070):736-47. doi: 10.1016/S0140-6736(16)30970-9

26. Traeger AC, Moseley GL, Hubscher M, et al. Pain education to prevent chronic low back pain: a study protocol for a randomised controlled trial. *BMJ Open* 2014;4(6):e005505. doi: 10.1136/bmjopen-2014-005505

27. Hurley J, O'Keeffe M, O'Sullivan P, et al. Effect of education on non-specific neck and low back pain: A meta-analysis of randomized controlled trials. *Man Ther* 2016;23:e1-2. doi: 10.1016/j.math.2016.02.009

28. Moseley GL. Evidence for a direct relationship between cognitive and physical change during an education intervention in people with chronic low back pain. *Eur J Pain* 2004;8(1):39-45. doi: 10.1016/S1090-3801(03)00063-6

29. Moseley GL, Nicholas MK, Hodges PW. A randomized controlled trial of intensive neurophysiology education in chronic low back pain. *Clin J Pain* 2004;20(5):324-30.

30. Moseley G, Butler D. Explain pain supercharged. *Adelaide, Australia: Noigroup publications* 2017

For peer review only - http://bmjopen.bmj.com/site/about/guidelines.xhtml

31. Sharma S, Pathak A, Jensen MP. Words that describe chronic musculoskeletal pain: implications for assessing pain quality across cultures. *J Pain Res* 2016;9:1057-66. doi: 10.2147/JPR.S119212 [published Online First: 2016/11/30]

32. Eldridge SM, Chan CL, Campbell MJ, et al. CONSORT 2010 statement: extension to randomised pilot and feasibility trials. *BMJ* 2016;355:i5239. doi: 10.1136/bmj.i5239

33. Chan AW, Tetzlaff JM, Gotzsche PC, et al. SPIRIT 2013 explanation and elaboration: guidance for protocols of clinical trials. *BMJ* 2013;346:e7586. doi: 10.1136/bmj.e7586

34. Chen WH, Revicki DA, Amtmann D, et al. Development and Analysis of PROMIS Pain Intensity Scale. *Quality of Life Research* 2012;20:18-18.

35. Bardin LD, King P, Maher CG. Diagnostic triage for low back pain: a practical approach for primary care. *Med J Aust* 2017;206(6):268-73.

36. Billingham SA, Whitehead AL, Julious SA. An audit of sample sizes for pilot and feasibility trials being undertaken in the United Kingdom registered in the United Kingdom Clinical Research Network database. *BMC Med Res Methodol* 2013;13:104. doi: 10.1186/1471-2288-13-104 [published Online First: 2013/08/22]

37. Mengoni SE, Gates B, Parkes G, et al. Wordless intervention for people with epilepsy and learning disabilities (WIELD): a randomised controlled feasibility trial. *BMJ Open* 2016;6(11):e012993. doi: 10.1136/bmjopen-2016-012993

38. Nepal Go. National Population and Housing Census 2011 (National Report). In: Statistics CBo, ed. Kathmandu: NHPC, 2012.

For peer review only

For peer review only - http://bmjopen.bmj.com/site/about/guidelines.xhtml

39. Yamato T, Maher C, Saragiotto B, et al. The TIDieR Checklist Will Benefit the Physical Therapy Profession. *J Orthop Sports Phys Ther* 2016;46(6):402-4. doi: 10.2519/jospt.2016.0108

40. Hoffmann TC, Glasziou PP, Boutron I, et al. Better reporting of interventions: template for intervention description and replication (TIDieR) checklist and guide. *BMJ* 2014;348:g1687. doi: 10.1136/bmj.g1687

41. Butler DS, Moseley GL. Explain Pain 2nd Edn: Noigroup Publications 2013.

42. Moseley GL, Butler DSB. Explain Pain Handbook: Protectometer2015.

43. Moseley GL, Butler DS. Fifteen Years of Explaining Pain: The Past, Present, and Future. *J Pain* 2015;16(9):807-13. doi: 10.1016/j.jpain.2015.05.005 [published Online First: 2015/06/09]

44. Clarke CL, Ryan CG, Martin DJ. Pain neurophysiology education for the management of individuals with chronic low back pain: A systematic review and meta-analysis. *Manual Ther* 2011;16(6):544-49. doi: 10.1016/j.math.2011.05.003

45. Traeger AC, Hubscher M, Henschke N, et al. Effect of Primary Care-Based Education on Reassurance in Patients With Acute Low Back Pain: Systematic Review and Meta-analysis. *JAMA Intern Med* 2015;175(5):733-43. doi: 10.1001/jamainternmed.2015.0217

46. Linton SJ, McCracken LM, Vlaeyen JW. Reassurance: help or hinder in the treatment of pain. *Pain* 2008;134(1-2):5-8. doi: 10.1016/j.pain.2007.10.002

47. Koes BW, van Tulder M, Lin CW, et al. An updated overview of clinical guidelines for the management of non-specific low back pain in primary care. *Eur Spine J* 2010;19(12):2075-94. doi: 10.1007/s00586-010-1502-y

For peer review only - http://bmjopen.bmj.com/site/about/guidelines.xhtml

48. Traeger AC, O'Hagan ET, Cashin A, et al. Reassurance for patients with non-specific conditions - a user's guide. *Braz J Phys Ther* 2017;21(1):1-6. doi: 10.1016/j.bjpt.2016.12.007

49. Stableford S, Mettger W. Plain language: a strategic response to the health literacy challenge. *J Public Health Policy* 2007;28(1):71-93. doi: 10.1057/palgrave.jphp.3200102

50. Bernstein IA, Malik Q, Carville S, et al. Low back pain and sciatica: summary of NICE guidance. *BMJ* 2017;356:i6748. doi: 10.1136/bmj.i6748

51. Group TOPTLBPW. Evidence-informed primary care management of low back pain: clinical practice guideline 2015 [Available from: http://www.topalbertadoctors.org/cpgs/885801.

52. Excellence NIfHaC. Low back pain and sciatica in over 16s: assessment and management 2016 [Available from: https://www.nice.org.uk/guidance/ng59.

53. Qaseem A, Wilt TJ, McLean RM, et al. Noninvasive Treatments for Acute, Subacute, and Chronic Low Back Pain: A Clinical Practice Guideline From the American College of Physicians. *Ann Intern Med* 2017;166(7):514-30. doi: 10.7326/M16-2367

54. O'Connell NE, Cook CE, Wand BM, et al. Clinical guidelines for low back pain: A critical review of consensus and inconsistencies across three major guidelines. *Best Practice & Research Clinical Rheumatology* 2017

55. National GCU. Low Back Pain and Sciatica in Over 16s: Assessment and Management. 2016

For peer review only - http://bmjopen.bmj.com/site/about/guidelines.xhtml

56. Chiarotto A, Deyo RA, Terwee CB, et al. Core outcome domains for clinical trials in non-specific low back pain. *Eur Spine J* 2015;24(6):1127-42. doi: 10.1007/s00586-015-3892-3

57. Geraghty AW, Stanford R, Little P, et al. Using an internet intervention to support self-management of low back pain in primary care: protocol for a randomised controlled feasibility trial (SupportBack). *BMJ Open* 2015;5(9):e009524. doi: 10.1136/bmjopen-2015-009524

58. Shanyinde M, Pickering RM, Weatherall M. Questions asked and answered in pilot and feasibility randomized controlled trials. *BMC Med Res Methodol* 2011;11:117. doi: 10.1186/1471-2288-11-117

59. Abbott JH. The distinction between randomized clinical trials (RCTs) and preliminary feasibility and pilot studies: what they are and are not. *J Orthop Sports Phys Ther* 2014;44(8):555-8. doi: 10.2519/jospt.2014.0110

60. Pinheiro MB, Ho KK, Ferreira ML, et al. Efficacy of a Sleep Quality Intervention in People With Low Back Pain: Protocol for a Feasibility Randomized Co-Twin Controlled Trial. *Twin Res Hum Genet* 2016;19(5):492-501. doi: 10.1017/thg.2016.67

61. Borkovec TD, Nau SD. Credibility of Analogue Therapy Rationales. *J Behav Ther Exp Psy* 1972;3(4):257-60. doi: Doi 10.1016/0005-7916(72)90045-6

62. Pathak A, Sharma S, Jensen MP. Comparing the psychometric properties of Nepali versions of four pain intensity rating scales in individuals with musculoskeletal pain. World Confederation for Physical Therapy Congress. Cape Town, South Africa, 2017.

For peer review only

For peer review only - http://bmjopen.bmj.com/site/about/guidelines.xhtml

63. Dworkin RH, Jensen MP, Gould E, et al. Treatment satisfaction in osteoarthritis and chronic low back pain: the role of pain, physical and emotional functioning, sleep, and adverse events. *J Pain* 2011;12(4):416-24. doi: 10.1016/j.jpain.2010.07.009

64. Dworkin RH, Turk DC, Farrar JT, et al. Core outcome measures for chronic pain clinical trials: IMMPACT recommendations. *Pain* 2005;113(1-2):9-19. doi: 10.1016/j.pain.2004.09.012 [published Online First: 2004/12/29]

65. Askew RL, Kim J, Chung H, et al. Development of a crosswalk for pain interference measured by the BPI and PROMIS pain interference short form. *Qual Life Res* 2013;22(10):2769-76. doi: 10.1007/s11136-013-0398-5

66. Sharma S, Pathak A, Maharjan R, et al. Psychometric properties of Nepali versions of PROMIS short form measures of pain intensity, pain interference, pain behavior, depression and sleep disturbance. . Poster will be presented at the 37th Annual Scientific Meeting (American Pain Society). Anaheim, (California), USA, 2018, March.

67. Sharma S, Thibault P, Abbott JH, et al. Clinimetric properties of the Nepali version of the Pain Catastrophizing Scale in individuals with chronic pain. *J Pain Res* 2018;11:265-76. doi: 10.2147/JPR.S153061 [published Online First: 2018/02/13]

68. Farrar JT, Young JP, Jr., LaMoreaux L, et al. Clinical importance of changes in chronic pain intensity measured on an 11-point numerical pain rating scale. *Pain* 2001;94(2):149-58. doi: S0304-3959(01)00349-9 [pii] [published Online First: 2001/11/03]

69. Sharma S, Palanchoke J, Reed D, et al. Translation, cross-cultural adaptation and psychometric properties of the Nepali versions of numerical pain rating scale and

For peer review only - http://bmjopen.bmj.com/site/about/guidelines.xhtml

global rating of change. *Health Qual Life Outcomes* 2017;15(1):236. doi: 10.1186/s12955-017-0812-8

70. Sharma S, Palanchoke J, Reed D, et al. Translation, cross-cultural adaptation and psychometric properties of the Nepali versions of Numerical Pain Rating Scale and Global Rating of Change. . *Submitted manuscript* 2017:In Review.

71. Crane HM, Van Rompaey SE, Dillingham PW, et al. A single-item measure of health-related quality-of-life for HIV-infected patients in routine clinical care. *AIDS Patient Care STDS* 2006;20(3):161-74. doi: 10.1089/apc.2006.20.161

72. Yu L, Buysse DJ, Germain A, et al. Development of short forms from the PROMIS sleep disturbance and Sleep-Related Impairment item banks. *Behav Sleep Med* 2011;10(1):6-24. doi: 10.1080/15402002.2012.636266 [published Online First: 2012/01/19]

73. Pilkonis PA, Choi SW, Reise SP, et al. Item banks for measuring emotional distress from the Patient-Reported Outcomes Measurement Information System (PROMIS(R)): depression, anxiety, and anger. *Assessment* 2011;18(3):263-83. doi: 10.1177/1073191111411667

74. Campbell-Sills L, Stein MB. Psychometric analysis and refinement of the Connor-davidson Resilience Scale (CD-RISC): Validation of a 10-item measure of resilience. *J Trauma Stress* 2007;20(6):1019-28. doi: 10.1002/jts.20271

75. Sharma S, Pathak A, Abbott JH, et al. Measurement properties of the Nepali version of the Connor Davidson Resilience Scales in individuals with chronic pain. *Health Qual Life Outcomes* 2018:Under Review.

For peer review only

For peer review only - http://bmjopen.bmj.com/site/about/guidelines.xhtml

Table 1. Schedule of enrolment, assessment, and interventions.

			Study period				
			Enrolment	Allocation	Post allocation		Final Assessment
		TIMEPOINT	-T_1	T_0	T_1	T_2	T_3
ENROLMENT		Eligibility screening	X				
		Explain study procedure/ provide participant information sheets	X				
		Informed consent	X				
		Random treatment allocation		X			
INTER-VENTION		Experimental intervention (PEG)				X	
		Control group (CG)				X	
Assessment	Baseline Descriptive Variables	Sociodemographic information (age, sex, address, occupation, religion, ethnicity.)			X		
	History	Pain history (site of pain, duration of pain, continuous or intermittent pain, aggravating and relieving factors)			X		
		Co-morbidities			X		
		Willingness to participate in a randomized controlled trial.	X				
	Feasibility outcomes	Acceptability of random allocation to one of the two groups	X				
		Acceptability of intervention session (one session in a week with home treatment program throughout the week)	X				
		*Feasibility of blinding the assessor					X
		Eligibility and recruitment rates	X				
		*Acceptability of screening procedures					W
		Understanding possible contamination between the groups					X
		Evaluating the credibility of the intervention			X		X
		Adherence to intervention					X
		Treatment satisfaction					X
		Difficulty in understanding the treatments					X
	Secondary outcomes	PROMIS Pain Interference			X		X
		PROMIS Pain intensity			X		X
		Quality of Life			X		X
		PROMIS Sleep disturbance			X		X
		PROMIS Depression			X		X
		GROC			-		X

For peer review only

For peer review only - http://bmjopen.bmj.com/site/about/guidelines.xhtml

		PCS			X		X
		CD-RISC-10			X		X

Note: *Assessed by the therapist providing intervention; all other outcomes are assessed by the blinded outcome assessor.

Abbreviations: T_1, Enrolment time; T_0, Allocation time; T_1, Baseline assessment (before treatment); T_2, During treatment; T_3, One week post-treatment; *PROMIS*, Patient-Reported Outcome Measurement Information System; GROC, Global Rating of Change; *PCS*, Pain Catastrophizing Scale; *CD-RISC-10*, 10-item Connor-Davidson Resilience Scale. *W*= assessment at the end of every week on Fridays.

For peer review only

For peer review only - http://bmjopen.bmj.com/site/about/guidelines.xhtml

Table 2. How will each primary feasibility objective be assessed?

SN	Objectives	Measures to assess specific objectives	Statistical analysis
1	Willingness to participate in a randomized controlled trial	All the consecutive participants presenting at the physiotherapy department will be approached invited to participate. The number and rate of participants willing versus not willing to participate will be recorded, as will the reason for not wanting to participate.	Total number of patients not willing to participate and reasons for non-participation will be summed.
2	Feasibility of blinding the assessor	Feasibility of blinding will be assessed by asking the assessor two questions at the end of the follow-up assessment:<br>1.Did you receive any information that indicated to you which group the participant was assigned to?<br>2.How did you receive information about group assignment?<br>Assessor's guess regarding group assignment group will be recorded for each participant. The responses will be coded as correct or incorrect guess.	The frequency and relative rates of "*Yes*" and "*No*" as the answer to the first question will be computed, as will the frequency and relative rates of correct and incorrect guesses. Finally, reasons for guesses will be recorded as verbatim and reported.
3	Eligibility and recruitment rates	The total number of participants screened, found eligible, and recruited will be recorded during the screening and recruitment process. The reasons why the participants were ineligible for study inclusion will be recorded for every participant who did not meet eligibility criteria. Reasons for declining participation will be recorded for every eligible individual who declined participation. Consent rates will also be recorded.	The number of participants screened, who were eligible, who consented to participate, who refused to participate, reasons for exclusion and refusal will be computed.
4	Acceptability of screening procedures	The lead researcher will interview the physiotherapist(s) screening the potential participants for eligibility by asking the following open-ended question at the end of every week, *"Were there any difficulties or challenges in screening and recruiting the participants the past week?"* The responses will be recorded as "Yes" or "No". If the answer to this question is "Yes", the lead researcher will ask the following open-ended question, "*What were the difficulties and challenges?*". Responses will be written down verbatim. Additionally, the outcome assessor will complete a form every time he/she encounters a difficulty or a challenge during screening. Outcome assessors will also be asked about their recommendations for overcoming any challenges that they	The frequency of difficulties or challenges will be counted.<br>The difficulties and challenges will be noted, categorized if possible, and reported. Results will be used to improve the screening procedure in the full trial in future, and therapists' recommendations will be considered.

For peer review only - http://bmjopen.bmj.com/site/about/guidelines.xhtml

		identify.<br>Time taken to complete the questionnaires will be recorded.	
5	Acceptability of random allocation to a treatment group	Outcome assessor will ask the participants if random allocation to one of the two treatment groups is acceptable to the participants. Responses will be recorded as "Acceptable", "Not acceptable", or "No preference".	The frequencies of each response will be computed separately for each treatment condition.
6	Understanding possible contamination between the groups	Contamination between the groups will be assessed by asking all the study participants the following questions at one week following treatment:<br>1. *"Have you talked to other participants in this study about the intervention they are receiving?"; If yes, further ask, "Was your attitude towards the intervention, or intervention changed after talking to one of the participants in the other group?";*<br>2. *"Are you aware of the intervention that any participants in the other group are receiving in the study?";*<br>3. *Are any of the participants in the other group aware of the type of intervention you are receiving in this study?"*<br>Participants in the usual care group will be asked the following question: "*Did you read the pain education booklet or watch the video which is one of the components of the intervention group*?"<br>These questions are adapted from a co-twin controlled feasibility trial.[60]	The frequencies of the participants who responded affirmatively to each question will be computed separately for each treatment condition.
7	Credibility and acceptability of the interventions	Treatment credibility will be assessed using five questions adapted from Borkovec and Nau.[61] The questions will be modified to fit "Pain education" and "Usual care" as a treatment for patients with LBP, and will be administered to all the participants in both conditions. One, important cultural adaptation for the scoring will be made by changing the numerical scale proposed by Borkovec and Nau to a Verbal Rating Scale, because Numerical Rating Scales have been shown to be unsuitable in Nepalese with musculoskeletal pain; especially in those who are older and have low educational attainment.[62] Responses for each of the five questions will be recorded on a Likert Scale where, 0= *"Not at all"*, 1= *"A little bit"*, 2= *"Somewhat"*, 3= *"Quite a bit"*, 4= *"Very much."*	Mean of the total scores on the credibility scale will be computed separately for each condition. Between-condition differences in creditability will be evaluated a *t*-test.

For peer review only - http://bmjopen.bmj.com/site/about/guidelines.xhtml

		The questions are:<br>**Questions:**<br>*1.How logical does pain education seem to you as a treatment?*<br>*2.How confident would you be that this treatment would be successful in reducing pain?*<br>*3.How confident would you be in recommending this treatment to a friend or family who also has LBP?*<br>*4.If you were having low back pain again, would you be willing to undergo such treatment?*<br>*5.How successful do you feel this treatment would be in reducing pain in other parts of the body, example, knee pain?*<br>All the questions will be asked at baseline and at one week following treatment.	
8	Adherence to the intervention	Adherence to treatment will be assessed by asking the participants in both conditions to maintain a record of daily home treatment that was followed by every participant. The participants will tick mark a box for every day for a total of five days between the two assessment timepoints to indicate their adherence to prescribed home exercise program. Additionally, participants will be requested to record any deviation of prescribed home treatment program (example, additional visit to the clinic and use of other interventions). Finally, question regarding non-adherence will be asked.	The total number of treatment adherence days will be summed for each treatment condition separately.<br>Deviation from the treatment protocol will be recorded.<br>Finally, the reasons for non-adherence will also be listed.
9	Satisfaction of treatment	All the participants in both the conditions will be asked to respond to the Patient Global Assessment of Treatment Satisfaction (PGATS) scale at one week following treatment.[63 64] The question asked will be, ''*How satisfied are you overall with the study treatment?*'' Responses to this question are made on a 5-point categorical scale (0 = ''*Very dissatisfied*''; 1 = ''*Dissatisfied*''; 2 = ''*Neutral or no preference*''; 3 = ''*Satisfied*''; 4 = ''*Very satisfied*'').	Mean treatment satisfaction scores will be compute for each treatment condition separately. Between-condition differences will be evaluated using a *t*-test.
10	Difficulty in understanding the information provided by the	All the participants will be asked about the difficulty in understanding the information provided by the physiotherapist. The question asked will be, "*How difficult was it for you to understand **<u>the information</u>** provided by the physiotherapist?*"	The differences in the difficulty in understanding the information provided will be compared between the two groups.

For peer review only - http://bmjopen.bmj.com/site/about/guidelines.xhtml

	physiotherapist.	Responses will be provided on a 5-point Likert Scale, where 1= "*Very easy*", 2= "*Easy*", 3= "*Neither easy nor difficult*", 4= "*Difficult*", 5= "*Very difficult*".	
11	Difficulty in understanding the instructions for what to do.	All participants will be asked about the difficulty in understanding the instructions provided by the physiotherapist for what to do at home. The question asked will be, "*How difficult was it for you to understand **the instructions** provided by the physiotherapist for what to do at home?*" using a on a 5-point Likert Scale, where 1= "Very easy", 2= "*Easy*", 3= "*Neither easy nor difficult*", 4= "*Difficult*", 5= "*Very difficult*".	Frequencies and rates of each response category will be computed for each group separately.
	Adverse events	All participants will be asked about any adverse events after treatment. All responses will be recorded verbatim.	The number of adverse events listed will be computed for each treatment condition separately. The responses will be analyzed qualitatively (see text).

For peer review only

For peer review only - http://bmjopen.bmj.com/site/about/guidelines.xhtml

Table 3. Secondary outcome measures.

Domain	Name of outcome measure	No. of Items	Response Scale	Scoring	Measurement properties of Nepali versions of the scale.
Pain interference	PROMIS Pain Interference short form 6b[65]	6	5 point, Ordinal	Respondents are asked to rate how much pain interfered with their daily life (e.g., enjoyment of life, ability to concentrate, day-to-day activities) in past 7 days on 5-point Likert scales (1 = "*Not at all*", 2 = "*A little bit*", 3 = "*Somewhat*", 4 = "*Quite a bit*", 5 = "*Very much*"). Responses are scored as a T-score that can range from 0-100, with a mean of 50 and SD of 10 in the normative sample.	• Good internal consistency (Cronbach's alpha= 0.85).<br>• Excellent reliability (ICC= 0.80) in a sample of individuals with chronic pain from Nepal.[66]
Pain catastrophizing	Pain Catastrophizing Scale (PCS)[67]	13	5-point, Ordinal	Respondents are asked to indicate the degree or frequency with which they have each catastrophizing response listed when they "… are experiencing pain" on a 5-point Likert scale (0 = "*Not at all*", 1 = "*To a slight degree*", 2 = "*To a moderate degree*", 3 = "*To a great degree*", and 4 = "*All the time*"). The total PCS score can range from 0 to 52, with higher scores representing higher pain catastrophizing.	• Good to excellent internal consistencies: Cronbach's alpha= 0.85- 0.93, and<br>• Excellent test-retest stability (ICC= 0.89- 0.90).<br>• Positive moderate correlations with measures of pain intensity, depression, and anxiety in a sample of individuals with chronic pain from Nepal.[67]
Patient's global rating of change	Global rating of Change (GROC)[64 68 69]	1	1-7, Ordinal	Respondents are asked to rate their "*overall improvement*" their health condition since the study enrolment. Responses can range from 1 to 7 with 4 = "*No change*". Scores greater than 4 indicate greater improvement and scores lower than 4 indicate a perceived worsening in the respondent's health condition.	MIC: 1 point change.[69 70]
Quality of life	Quality-of-Life (QOL) rating	2	5-point, Ordinal	Respondents are asked to rate their general quality of life and general health by responding	Not available during the time of protocol writing the study protocol.

For peer review only - http://bmjopen.bmj.com/site/about/guidelines.xhtml

	scale			to the question, "*In general, how would you rate your overall quality of life during the past week?*" The response options are 1 = "Very bad", 2= "Bad", 3= "Fair", 4= "Good", and 5= "Very good".[71] Greater score indicate better quality of life.	
Pain intensity	PROMIS Pain Intensity short form 3b	3	5-point, Ordinal	Respondents are asked to rate their current pain, and their worst and average pain intensity in past 7 days on 5-point Likert Scales (1 = "*Had no Pain*", 2 = "*Mild*", 3 = "*Moderate*", 4 = "*Severe*", and 5 = "*Very severe*"). Responses are scored as a T-score that can range from 0-100, with a mean of 50 and SD of 10 in the normative sample.	Good test-retest reliability (Intraclass Correlation Coefficient [ICC]= 0.71) in a sample of individuals with chronic pain from Nepal.[66]
Sleep disturbance	PROMIS Sleep Disturbance short form 8b[72]	7*	5-point, Ordinal	Respondents are asked to rate sleep quality items on a 5-point Likert scale. Responses are scored as a T-score that can range from 0-100, with a mean of 50 and SD of 10 in the normative sample.	Although the Nepali translation of the 8-item Sleep Disturbance short-form showed poor reliability, removal of one item, improved the test-retest stability to excellent. (ICC= 0.78).<br>Good internal consistency of 7-items (Cronbach's alpha = 0.89).[66]
Depression	PROMIS Emotional Distress-Depression-short form 8b[73]	8	5-point, Ordinal	Respondents are asked to indicate the frequency of depressive symptoms in the past 7 days on a 5-point verbal rating scale (1 = "*Never*", 2 = "*Rarely*", 3 = "*Sometimes*", 4 = "*Often*", and 5 = "*Always*"). Responses are scored as a T-score that can range from 0-100, with a mean of 50 and SD of 10 in the normative sample.	• Excellent internal consistency (Cronbach's alpha= 0.93).<br>• Excellent test-retest reliability (ICC= 0.81) in a sample of individuals with chronic pain from Nepal.[66]
Resilience	10-item Connor Davidson Resilience Scale[74]	10	4-point, Ordinal	Respondents are asked to rate each resiliency item on a 5-point Likert scale: 0 = "*Not true at all*", 1 = "*Rarely true*", 2 = "*Sometimes true*", 3 = "*Often true*", and 4 = "*True nearly all the time*". Responses are summed such that total scores range from 0 to 40, with higher scores indicating	• Good to excellent internal consistency (Cronbach's alpha= 0.87- 0.90).[75]<br>• Excellent test-retest stability (ICC=0.89).[66]<br>• Standard error of measurement= 2.42 points.

For peer review only - http://bmjopen.bmj.com/site/about/guidelines.xhtml

				more resilience.	• Minimum detectable change= 6.72 points.<br>• Significant negative and moderate association with the PCS in a sample of individuals with chronic pain from Nepal.[66]
Use of pain medications and other pain treatments	-	-	-	Names, and dosage of pain medication intake will be recorded by the assessor by asking the research participant via interview. Medications will then be categorized into analgesic type (opioids, NSAIDs, sedatives, and anti-seizure medications), as will the number of days each type of medication is taken.<br>Other pain treatments provided to or used by the study participants will also be recorded and classified (e.g., physical therapy, naturopathy). The number of days each treatment was provided to or used by the participants will be recorded.	• No validity data for self-reported analgesic or pain treatment use in Nepali patients available at the time of protocol writing.

*Only seven items out of 8 items will be included, as the measurement properties of the total 8-item was poor, and only after removal of 1 item, the reliability improved.

For peer review only - http://bmjopen.bmj.com/site/about/guidelines.xhtml

Table 4. Criteria for feasibility.

	Full trial is not possible as designed		Proceed to a full trial without modification in the protocol of the feasibility trial	
**Criteria**	Modify the protocol prior to a full trial if…	Action	Monitor the study procedures closely if…	Close monitoring is not required if…
**Blinding of assessor**	Assessor has a >70% correct guess rate on the group allocation.	Identify ways to improve assessor blinding based on the responses or feedback provided by the assessors.	70-90% blinding is found.	<10% incorrect guess to the group allocation.
**Recruitment rate**	≤1 participant every week	Identify reasons for low participation rates or declining participation.<br>Possible strategies could be changing the study site, increasing the number of study sites, increasing advertising efforts, using incentives for participation.	2-3 participants recruited every week.	≥ 4 participants recruited every week.
**Attrition rate (in both arms)**	>30% total drop-outs at 1 week post-treatment.	Identify possible reasons for drop-outs and ways to improve follow-up participation.	15%-30% total drop-outs at 1 week post-treatment.	<15% total drop-outs at 1 week post-treatment. (www.pedro.org.au)
**Feasibility of outcome assessment**	>20% missing data on the secondary outcome measures.	Reduce the number of outcome measures.<br>Identify and use brief versions of outcomes.	10%-20% missing data on the secondary outcome measures.	<10% missing data on the secondary outcome measures.
**Contamination of intervention**[60]	≥ 15% contamination between the groups.	Identify reasons for contamination and resolve them.	<15% contamination between the groups.	0% contamination between the groups.
**Score on the credibility of treatment scale**	Control condition is > .50 SD less credible that EP.	Develop a new control condition for the EP trial, and pilot test to ensure that its credibility rating is < .50 SD different from EP.	-	The credibility scores of the two conditions are w/i .50 SD units of each other.
**Adherence to treatment**	< 50% attend the treatment. session after randomization.	Identify reasons for not attending the treatment session in order to increase attendance in the full trial.	50%-80% of the participants attend the treatment session.	80% or more of the participants attend the treatment sessions.

For peer review only - http://bmjopen.bmj.com/site/about/guidelines.xhtml

**Difficulty scale**	If ≥50% participants in the experimental group rate pain education as very difficult, very easy, easy, or neither easy nor difficult.	Make the intervention a little more challenging if more than half participants report 1-3 out of 5 in the difficulty scale, by increasing the depth of the education, by adding more pain biology education content. Whereas, if more than half participants report the intervention as very difficult, reduce the complexity. Re-evaluate complexity in a second cohort of at least five participants to ensure appropriate level of difficulty.	-	≥50% participants in the experimental group rate pain education as a “difficult” intervention (4 out of 5 in the difficulty scale). This is the preferred difficulty level because we want pain education intervention to be difficult enough to challenge participants – but not too easy or very difficult.

For peer review only - http://bmjopen.bmj.com/site/about/guidelines.xhtml
For peer review only

Explaining Pain in an hour

Curriculum for Nepalese with non-specific low back pain

Learner or patient characteristics

Learners are adult patients who present to a Physiotherapy Department in Kathmandu, Nepal for the management of their low back pain (LBP). This education will be delivered to patients with any duration of LBP. Every participant who understands and can speak Nepali can be the learner for the planned curriculum of Explain Pain. These individuals may have a very low literacy level. Patients who have diagnosed psychiatric illness will be excluded from this intervention.

Deliverer or physiotherapist

A physiotherapist (Saurab Sharma) who has undergone “Explain Pain” course twice (in 2015 and 2017) will deliver Explain Pain to all the participants in the study. The physiotherapist will be supervised by Prof. G. Lorimer Moseley, one of the developers of the concept.

Number of learners or patients

A total of 40 learners will participate in Explain Pain in this study. All interventions will be delivered individually to each participant. No other concurrent interventions will be provided to the patients with Explain Pain, however, patients will be encouraged to perform some exercises at home for one week.

Unique needs of the learners

These learners are from Nepal, many of them may not have any formal education, and education as the core component of treatment may be new to these patients. Thus, this group may be a challenging group to provide Explain Pain intervention. Therefore, the contents are simplified and adapted to their need to match their level of understanding, and culture.

Delivery methods

The study physiotherapist will deliver approximately an hour long session of one-on-one Explain Pain to every study participant. Every participant will be provided with a take away education booklet, which includes details of target concept, pictures and stories to strengthen their education provided by the physiotherapist face-to-face. Participants will read the booklet if they are educated, or the family members can read out for them if they cannot read themselves. All the participants will be provided with an additional audio-visual information on neurophysiology knowledge of pain, if they can operate these at home (or office) to reinforce the learning so that the participants hear this every day before the follow-up for assessment in a week.

Place

For peer review only - http://bmjopen.bmj.com/site/about/guidelines.xhtml

A room at a physiotherapy facility (Sahara Care Hospital) in Kathmandu, Nepal.

Patient consideration

1. Physiotherapist will extract learner's personal goal of treatment. Nepalese generally struggle to bring out their own goals in general, this will be a difficult task.
2. Involvement of family throughout the education session will be encouraged if they accompany the patients. Role of family members will be highlighted in the education program and prognosis of the individuals with LBP.
3. All the participants will be reminded to perform their home-based tasks (reading, listening/watching audio-visual support, and exercising) by a text message or a phone call every day for five days a week.

Aims

The deliverer intends to:

1. Advise patient that educate is an important aspect of treatment of pain.
2. Provide contemporary knowledge about pain biology in relation to low back pain in an individual face-to-face teaching and learning environment.
3. Provide a comprehensive patient education by providing relevant information on graded exposure, pacing, and self-management.
4. Use pictures, metaphors, and relevant stories related to pain to explain details and complexities related to pain.

Objectives

At the end of the session learner will:

1. Have contemporary knowledge of pain biology that is relevant to their low back pain.
2. Understand importance of pain knowledge as a therapy.
3. Use the pain knowledge in changing the danger messages into safety signals, and using exercises to pave the track for recovery.

For peer review only - http://bmjopen.bmj.com/site/about/guidelines.xhtml

Explain Pain Curriculum plan

Part 1: Question and answer **[10 – 15 minutes]**

Physiotherapist will first toss two questions.

Question 1- "*Is there anything in particular that you would like to learn about your low back pain, or pain in general?*"

Physiotherapist will answer to any questions that arise.

Question 2- *"Do you know what caused your low back pain? Can you please explain the cause of your low back pain from what you have understood, or what you have been told?"*

Address any misconceptions and acknowledge and appreciate healthy/sound understanding about their pain. Talk about scans if appropriate.

Part 2: Discuss the key concepts of pain biology. **[40 minutes]**

1. Pain is normal and almost everyone gets it in life.
2. Body sends danger signals, and brain decides whether to produce pain.
3. Learning about pain changes pain; and anything associated with it can influence it.
4. Body learns pain and becomes overprotective over time.
5. Additional concept: Pain and tissue damage are poorly related.

[see the table below for the details]

Part 3: Do you want to learn ways to train your system? **[5 – 10 minutes]**

Teach 1 – 2 ways to train the system.

End: Ask if the patient has a cell phone. If he or she has a cell phone, ask if he/she wants to receive a daily reminder to perform home-based tasks, and learn more about pain.

Record the number and send the information daily.

For peer review only - http://bmjopen.bmj.com/site/about/guidelines.xhtml

Explain Pain Curriculum Plan

SN	Target concepts	Time required	Other ways of expressing the target concept	Content	Delivery and resources	Reinforcement/ Experiential learning	Did the patient understand? Assessment
1	Pain is normal and almost everyone gets it in life.	10 minutes	• Everyone has some pain in lifetime so you are not alone.<br>• Pain is normal.<br>• Your LBP is yours, is unique to you and real, and only you can control your pain.	• There is no test for pain or love.<br>• Emotional and physical pain are one.<br>• Pain is always a conscious event.	• Stories<br>• Brief pain epidemiology	• Pain should not be a reason to worry about, and stop you from enjoying life, and fulfilling life goals.	• Ask- so who suffers pain or how many people suffer pain?<br><br>*Answer- almost everyone.*
2	Body sends danger signals, and brain decides whether to produce pain.	10 minutes	• How danger signal travels in the body and how pain is perceived.<br>• Brain is needed to create/ perceive pain.<br>• Human body has danger sensors not pain sensors.<br>• Pain depends on the balance between danger and safety.	• Nociceptive pathway.<br>• Pain is created in the brain.<br>• Ask patient if they had pain when they did not have tissue injury (or use aggravating factors).	• Use a picture/ animation of how danger signals reach brain?<br><br>• Use the earthquake story.	• Have you ever experienced having an injury and no pain?<br>• Have you ever experienced pain when there was no injury?<br>• Use the scale protectometer to describe.	• So what creates pain?<br><br>*Answer: brain!!!*
3	Learning about pain changes pain; and anything associated with it can influence it.	10 minutes	• Knowing about pain can reduce pain.<br>• Education is analgesic.<br>• Retraining your system can reduce sensitization.	• Understanding your pain can reduce your pain.<br>• Wrong understanding can increase your pain.	• Sikha's pain story.	• Hear other's pain stories and analyse how it can be changed.	
4	Body learns pain and becomes	10 minutes	• Out of many outputs of the brain, pain is only one	• As pain persists, body becomes sensitized and	• The bending and lifting	• Ask the patient what are the	• Ask, do you understand this

For peer review only - http://bmjopen.bmj.com/site/about/guidelines.xhtml

	overprotective over time.		protective output.<br>• As pain persists, body systems can be over protective.<br>• Multiple systems protect us from threats, and allow us to learn and heal.<br>• You can train your body systems to be less protective.	over protective.<br>• This can be changed by training our systems.	story.	other symptoms they get with pain? [Examples are: sweating, no sleep, stress, anxiety, fear, anger etc].	and think if this is logical?<br>• Reinforce this by saying, this is also scientific.
Optional	Pain and tissue damage are poorly related	5 minutes	• Pain is an unreliable indicator of tissue damage.<br>• Pain and scans do not correlate.	• Tissue stop hurting a long time before they heal.<br>• Recent evidences regarding poor correlation between pain and scan reports.	Bad scans in pain free people.	Reinforce by saying degeneration is like greying of hair, which is normal ageing process. It does not hurt.	

For peer review only

STANDARD PROTOCOL ITEMS: RECOMMENDATIONS FOR INTERVENTIONAL TRIALS

SPIRIT 2013 Checklist: Recommended items to address in a clinical trial protocol and related documents*

Section/item	Item No	Description	Addressed on page number
**Administrative information**			
Title	1	Descriptive title identifying the study design, population, interventions, and, if applicable, trial acronym	_____1________
Trial registration	2a	Trial identifier and registry name. If not yet registered, name of intended registry	_____3________
	2b	All items from the World Health Organization Trial Registration Data Set	______________
Protocol version	3	Date and version identifier	______________
Funding	4	Sources and types of financial, material, and other support	_____22______
Roles and responsibilities	5a	Names, affiliations, and roles of protocol contributors	_____1, 22____
	5b	Name and contact information for the trial sponsor	_____N/A_____
	5c	Role of study sponsor and funders, if any, in study design; collection, management, analysis, and interpretation of data; writing of the report; and the decision to submit the report for publication, including whether they will have ultimate authority over any of these activities	_____N/A_____
	5d	Composition, roles, and responsibilities of the coordinating centre, steering committee, endpoint adjudication committee, data management team, and other individuals or groups overseeing the trial, if applicable (see Item 21a for data monitoring committee)	_____20-21_____

For peer review only - http://bmjopen.bmj.com/site/about/guidelines.xhtml

Introduction

Background and rationale	6a	Description of research question and justification for undertaking the trial, including summary of relevant studies (published and unpublished) examining benefits and harms for each intervention	____4-6_____
	6b	Explanation for choice of comparators	______5______
Objectives	7	Specific objectives or hypotheses	________7_____
Trial design	8	Description of trial design including type of trial (eg, parallel group, crossover, factorial, single group), allocation ratio, and framework (eg, superiority, equivalence, noninferiority, exploratory)	______6______

Methods: Participants, interventions, and outcomes

Study setting	9	Description of study settings (eg, community clinic, academic hospital) and list of countries where data will be collected. Reference to where list of study sites can be obtained	______7-8_____
Eligibility criteria	10	Inclusion and exclusion criteria for participants. If applicable, eligibility criteria for study centres and individuals who will perform the interventions (eg, surgeons, psychotherapists)	______9-11____
Interventions	11a	Interventions for each group with sufficient detail to allow replication, including how and when they will be administered	______11-17___
	11b	Criteria for discontinuing or modifying allocated interventions for a given trial participant (eg, drug dose change in response to harms, participant request, or improving/ worsening disease)	______N/A____
	11c	Strategies to improve adherence to intervention protocols, and any procedures for monitoring adherence (eg, drug tablet return, laboratory tests)	______17______
	11d	Relevant concomitant care and interventions that are permitted or prohibited during the trial	______11-17___
Outcomes	12	Primary, secondary, and other outcomes, including the specific measurement variable (eg, systolic blood pressure), analysis metric (eg, change from baseline, final value, time to event), method of aggregation (eg, median, proportion), and time point for each outcome. Explanation of the clinical relevance of chosen efficacy and harm outcomes is strongly recommended	_18, 35-43____
Participant timeline	13	Time schedule of enrolment, interventions (including any run-ins and washouts), assessments, and visits for participants. A schematic diagram is highly recommended (see Figure)	____33-34_____

For peer review only - http://bmjopen.bmj.com/site/about/guidelines.xhtml

Sample size	14	Estimated number of participants needed to achieve study objectives and how it was determined, including clinical and statistical assumptions supporting any sample size calculations	_____10______
Recruitment	15	Strategies for achieving adequate participant enrolment to reach target sample size	____10-11_____

Methods: Assignment of interventions (for controlled trials)

Allocation:

Sequence generation	16a	Method of generating the allocation sequence (eg, computer-generated random numbers), and list of any factors for stratification. To reduce predictability of a random sequence, details of any planned restriction (eg, blocking) should be provided in a separate document that is unavailable to those who enrol participants or assign interventions	_____11______
Allocation concealment mechanism	16b	Mechanism of implementing the allocation sequence (eg, central telephone; sequentially numbered, opaque, sealed envelopes), describing any steps to conceal the sequence until interventions are assigned	_____11______
Implementation	16c	Who will generate the allocation sequence, who will enrol participants, and who will assign participants to interventions	______11______
Blinding (masking)	17a	Who will be blinded after assignment to interventions (eg, trial participants, care providers, outcome assessors, data analysts), and how	______18______
	17b	If blinded, circumstances under which unblinding is permissible, and procedure for revealing a participant's allocated intervention during the trial	_____N/A______

Methods: Data collection, management, and analysis

Data collection methods	18a	Plans for assessment and collection of outcome, baseline, and other trial data, including any related processes to promote data quality (eg, duplicate measurements, training of assessors) and a description of study instruments (eg, questionnaires, laboratory tests) along with their reliability and validity, if known. Reference to where data collection forms can be found, if not in the protocol	18, 19, 35-41
	18b	Plans to promote participant retention and complete follow-up, including list of any outcome data to be collected for participants who discontinue or deviate from intervention protocols	____18_________

For peer review only - http://bmjopen.bmj.com/site/about/guidelines.xhtml

Data management	19	Plans for data entry, coding, security, and storage, including any related processes to promote data quality (eg, double data entry; range checks for data values). Reference to where details of data management procedures can be found, if not in the protocol	____21________
Statistical methods	20a	Statistical methods for analysing primary and secondary outcomes. Reference to where other details of the statistical analysis plan can be found, if not in the protocol	_21-22, 36-39__
	20b	Methods for any additional analyses (eg, subgroup and adjusted analyses)	_____N/A_____
	20c	Definition of analysis population relating to protocol non-adherence (eg, as randomised analysis), and any statistical methods to handle missing data (eg, multiple imputation)	______N/A____
**Methods: Monitoring**			
Data monitoring	21a	Composition of data monitoring committee (DMC); summary of its role and reporting structure; statement of whether it is independent from the sponsor and competing interests; and reference to where further details about its charter can be found, if not in the protocol. Alternatively, an explanation of why a DMC is not needed	_____20-21_____
	21b	Description of any interim analyses and stopping guidelines, including who will have access to these interim results and make the final decision to terminate the trial	____N/A______
Harms	22	Plans for collecting, assessing, reporting, and managing solicited and spontaneously reported adverse events and other unintended effects of trial interventions or trial conduct	_____19________
Auditing	23	Frequency and procedures for auditing trial conduct, if any, and whether the process will be independent from investigators and the sponsor	_____N/A______
**Ethics and dissemination**			
Research ethics approval	24	Plans for seeking research ethics committee/institutional review board (REC/IRB) approval	_____3, 22_____
Protocol amendments	25	Plans for communicating important protocol modifications (eg, changes to eligibility criteria, outcomes, analyses) to relevant parties (eg, investigators, REC/IRBs, trial participants, trial registries, journals, regulators)	______3_______

For peer review only - http://bmjopen.bmj.com/site/about/guidelines.xhtml

Consent or assent	26a	Who will obtain informed consent or assent from potential trial participants or authorised surrogates, and how (see Item 32)	_______11_______
	26b	Additional consent provisions for collection and use of participant data and biological specimens in ancillary studies, if applicable	_______N/A______
Confidentiality	27	How personal information about potential and enrolled participants will be collected, shared, and maintained in order to protect confidentiality before, during, and after the trial	_______21______
Declaration of interests	28	Financial and other competing interests for principal investigators for the overall trial and each study site	_______22______
Access to data	29	Statement of who will have access to the final trial dataset, and disclosure of contractual agreements that limit such access for investigators	_______N/A_____
Ancillary and post-trial care	30	Provisions, if any, for ancillary and post-trial care, and for compensation to those who suffer harm from trial participation	______N/A_______
Dissemination policy	31a	Plans for investigators and sponsor to communicate trial results to participants, healthcare professionals, the public, and other relevant groups (eg, via publication, reporting in results databases, or other data sharing arrangements), including any publication restrictions	_______3________
	31b	Authorship eligibility guidelines and any intended use of professional writers	_______22_______
	31c	Plans, if any, for granting public access to the full protocol, participant-level dataset, and statistical code	________N/A_____
**Appendices**			
Informed consent materials	32	Model consent form and other related documentation given to participants and authorised surrogates	______Yes_______
Biological specimens	33	Plans for collection, laboratory evaluation, and storage of biological specimens for genetic or molecular analysis in the current trial and for future use in ancillary studies, if applicable	______N/A______

*It is strongly recommended that this checklist be read in conjunction with the SPIRIT 2013 Explanation & Elaboration for important clarification on the items. Amendments to the protocol should be tracked and dated. The SPIRIT checklist is copyrighted by the SPIRIT Group under the Creative Commons "Attribution-NonCommercial-NoDerivs 3.0 Unported" license.

For peer review only - http://bmjopen.bmj.com/site/about/guidelines.xhtml

BMJ Open

Pain Education for patients with non-specific low back pain in Nepal: Protocol of a feasibility randomized clinical trial (PEN-LBP Trial).

Journal:	*BMJ Open*
Manuscript ID	bmjopen-2018-022423.R1
Article Type:	Protocol
Date Submitted by the Author:	23-May-2018

Complete List of Authors:	Sharma, Saurab; Kathmandu University School of Medical Sciences, Department of Physiotherapy; University of Otago Dunedin School of Medicine, Orthopedic Surgery Section, Department of Surgical Sciences<br>Jensen, Mark; University of Washington, Department of Rehabilitation Medicine<br>Moseley, G.; Sansom Institute for Health Research, University of South Australia<br>Abbott, J. Haxby; University of Otago, Department of Surgical Sciences, Dunedin School of Medicine

**Primary Subject Heading**:	Evidence based practice
Secondary Subject Heading:	Global health, Epidemiology
Keywords:	Explain pain, Physiotherapy, Low-income country, Low back pain, Patient education, Back pain < ORTHOPAEDIC & TRAUMA SURGERY

SCHOLARONE™
Manuscripts

Title: Pain Education for patients with non-specific low back pain in Nepal: Protocol of a feasibility randomized clinical trial (PEN-LBP Trial).

Authors: Saurab Sharma [1, 2]

Mark P. Jensen [3]

Lorimer G. Moseley [4]

J. Haxby Abbott [2]

Affiliations: [1] Department of Physiotherapy, Kathmandu University School of Medical Sciences, Dhulikhel, Nepal

[2] Centre for Musculoskeletal Outcomes Research, Dunedin School of Medicine, University of Otago, Dunedin, New Zealand

[3] Department of Rehabilitation Medicine, University of Washington, Seattle, USA

[4] School of Health Sciences, University of South Australia, Adelaide, Australia.

Present address of corresponding author:

Saurab Sharma
Assistant Professor, Department of Physiotherapy
Kathmandu University School of Medical Sciences
Dhulikhel Hospital Kathmandu University Hospital
Dhulikhel, P.O. Box No.: 11008, Nepal
Office fax: +977 1 490 707
saurabsharma1@gmail.com
Phone: +977 984 163 4043

For peer review only - http://bmjopen.bmj.com/site/about/guidelines.xhtml

ABSTRACT

Introduction: Low back pain (LBP) is the leading cause of years lived with disability in Nepal and elsewhere. Management of LBP that is evidence-based, easily accessible, cost-effective, and culturally appropriate is desirable. The primary aim of this feasibility randomized clinical trial is to determine if it is feasible to conduct a full randomized clinical trial evaluating the effectiveness of pain education as an intervention for patients in Nepal with LBP, relative to guideline-based physiotherapy treatment. The findings of the study will inform the planning of a full clinical trial, and if any modifications are required to the protocol before undertaking a full trial.

Methods/analysis: This protocol describes an assessor-blinded feasibility clinical trial investigating feasibility of the Pain Education intervention in patients with non-specific LBP in a physiotherapy hospital in Kathmandu, Nepal. Forty patients with LBP will be randomly allocated to either Pain Education or guideline-based physiotherapy treatment (control). Outcomes will be assessed at baseline and at a one-week post-treatment. The primary outcomes are related to feasibility, including: (1) participant willingness to participate in a randomized clinical trial, (2) feasibility of assessor blinding, (3) eligibility and recruitment rates, (4) acceptability of screening procedures and random allocation, (5) possible contamination between the groups, (6) intervention credibility, (7) intervention adherence, (8) treatment

For peer review only

satisfaction, and (9) difficulty in understanding the interventions being provided.

Ethics/dissemination: The protocol was approved by Nepal Health Research Council (NHRC; registration number: 422/2017) and University of Otago Human Ethics Committee for Health (registration number: H17/157). The results of the study will be presented at national and international conferences, and published in a peer-reviewed journal.

Trial registration: ClinicalTrials.gov: NCT03387228.

Keywords: Explain pain; Explaining pain; Back pain; Physiotherapy; Low-income country; Developing country

Strengths and limitations of this study

- Assessor and statistician will be blinded to group allocation of the participants.
- Therapist and patient blinding is not possible in this study because of the nature of the treatments provided.
- Conclusions regarding the effectiveness of the intervention cannot be made because of the feasibility design of the current study.

For peer review only - http://bmjopen.bmj.com/site/about/guidelines.xhtml

INTRODUCTION

Low back pain (LBP) is a highly prevalent health condition worldwide.[1 2] It is the leading cause of disability,[2] and imposes huge economic burden to the society in both developed and developing countries.[3-6] LBP is among the most common health conditions contributing to years lived with disability in Nepal.[2] Although the prevalence of LBP is high in Nepal, ethnographic research has noted that LBP-related disability may be low in rural areas,[7] perhaps due to the very low socioeconomic status of individuals living in rural Nepal, which forces them to keep working despite the presence of pain. Consistent with this idea, another study highlighted that 80% of people with chronic pain in Nepal continue to work.[6] However, it is alarming that number of spine surgeries for spinal pain has been increasing in Nepal over the years,[8] despite lack of evidence supporting efficacy for this treatment.[1 9]

Interventions for management of LBP

Many interventions have been investigated for the management of LBP. These include surgery,[10 11] pharmacotherapy,[12-17] exercises,[18-20] advice for self-management including advice to remain physically active,[11] and psychological therapies.[21 22] As alluded to earlier, biomedically-focused interventions such as surgery and pharmacotherapy are not recommended for a non-specific LBP as the evidence does not support their effectiveness.[1 14] Moreover, they are associated with significant risks for adverse events and are costly.[5]

For peer review only - http://bmjopen.bmj.com/site/about/guidelines.xhtml

Clinical practice guidelines for LBP recommend self-management including reassurance, education and advice to remain active as the first line of care that should be provided to all the patients with LBP. Superficial heat and manual therapy (massage/manipulative therapy) are recommended for acute LBP, whereas exercise and psychological therapies are recommended for chronic LBP.[23-25]

Pain education for LBP

Patient education for LBP that has been investigated in RCTs are basically of two types: biomedical education and pain biology education.[26] The first refers to educating patients about vertebral anatomy and patho-anatomy of the spine, which has been shown to be ineffective and may even have negative effects on LBP outcomes .[26] On the other hand, the second type of education – pain biology education (hereafter called as "pain education") has been shown to have positive effects on both pain and disability.[27 28] Pain education is structured education program with specific aims and objectives.[29] This intervention has a list of target key concepts to be delivered and includes the curriculum contents to deliver the key concepts using up-to-date pain science knowledge, stories, and metaphors.

It has been previously hypothesized that this type of education program utilizing metaphors and stories may be an effective intervention in Nepalese with chronic pain.[30] However, the pain education materials that have been developed in western cultures are not necessarily valid and equally effective in reducing pain and disability in non-western cultures.

For peer review only - http://bmjopen.bmj.com/site/about/guidelines.xhtml

Therefore, when developing pain education materials in a newer language or culture, (significant) cultural adaptations of the education materials may be required to make it suitable for the target population, as culturally inappropriate education may not produce desirable results.

Therefore, in order to evaluate the effectiveness of pain education in individuals with non-specific LBP from Nepal, culturally-appropriate pain education materials should first be developed for Nepal, specifically. However, it is possible that the adaptations made could potentially reduce its effectiveness. Thus, before testing the adapted pain education in a full clinical trial, a feasibility study is needed in order to determine if a full clinical trial based on the adapted intervention is warranted, or if additional modifications may be needed prior to performing the full trial.

Why the feasibility trial?

We propose a feasibility trial because: (1) the intervention (i.e., pain education) will need significant cultural adaptation, although it has been evaluated for efficacy previously in other languages and western cultures; (2) the adapted intervention has never been investigated for its efficacy or effectiveness before; (3) the population in question (individuals with extremely low socioeconomic status and educational attainment in Nepal) is unique; and (4) a high quality clinical trial in individuals with LBP has not been conducted in Nepal, to our knowledge, and we therefore do not know if a full trial is feasible. The findings from the proposed feasibility study will

For peer review only - http://bmjopen.bmj.com/site/about/guidelines.xhtml

inform the planning and design of a full trial, if the results indicate that a full trial is warranted.

The results of the full trial will have significant clinical implications for the management of LBP in Nepal, and similar cultures, providing empirical evidence if pain education is a viable treatment option for the management of LBP, and if it is effective in reducing pain, disability, and emotional distress.

Aims and objectives

The primary aim of this feasibility randomized study is to evaluate the feasibility of a full randomized clinical trial (as designed in this protocol) for assessing the effects of pain education as an intervention for patients with LBP of any duration in a physiotherapy facility in Nepal after developing culturally-appropriate, evidence-based pain education materials. The primary objectives of the study are related to feasibility of randomized clinical trial, specifically: (1) willingness to participate in a randomized controlled trial, (2) feasibility of assessor blinding, (3) eligibility and recruitment rates, (4) acceptability of screening procedures and random allocation, (5) possible contamination between the groups, (6) intervention credibility for patients with LBP, (7) intervention adherence, (8) treatment satisfaction, and (9) difficulty in understanding the intervention being provided.

For peer review only - http://bmjopen.bmj.com/site/about/guidelines.xhtml

METHODS AND ANALYSIS

Study Design and setting

This is a feasibility study that is being performed to determine if a full randomized controlled trial (RCT) can be successfully conducted using the procedures and protocol of the feasibility study, or if modifications of the feasibility study protocol are needed prior to conducting the full trial. The study findings will inform the design of the full trial, if a full trial is found to be feasible.[31]

The definition of a feasibility study highlights the question, *"Can this study be done?"* The SPIRIT statement,[32] the CONSORT statement extension to pilot and feasibility randomized trial,[31] was followed in the planning of the study and reporting of the protocol.

The study will be an assessor-blinded, two-arm, feasibility randomized clinical trial (RCT). Ethical approval has been obtained from the Nepal Health Research Council (NHRC, reference number: 422/2017) and University of Otago Human Ethics Committee (reference number: H17/157). The study is registered in Clinicaltrials.gov (trial registration number: NCT03387228). The study will be conducted in the Sahara Physiotherapy Hospital, Kathmandu, Nepal.

Overview of the study

For peer review only - http://bmjopen.bmj.com/site/about/guidelines.xhtml

Advertisement of the trial will be made in social media and all the patients with LBP presenting at the study site will be invited to participate. Interested candidates will be screened for eligibility. Eligible patients with non-specific LBP will then be enrolled in the trial and be randomly assigned to one of the two study groups. All the participants in the experimental group will receive pain education and those in the control group will receive evidence based physiotherapy treatment. All the participants will be assessed at baseline, and one week following treatment. Details describing the schedule of enrolment, interventions, and assessment are presented in Table 1, in the manner recommended by SPIRIT checklist.[32]

Table 1. Schedule of enrolment, assessment, and interventions.

			Study period				
			Enrolment	Allocation	Post allocation		Final Assessment
		TIMEPOINT	$-T_1$	T_0	T_1	T_2	T_3
ENROLMENT		Eligibility screening	X				
		Explain study procedure/ provide participant information sheets	X				
		Informed consent	X				
		Random treatment allocation		X			
INTER-VENTION		Experimental intervention (PEG)				X	
		Control group (CG)				X	
Assessment	Baseline Descriptive Variables	Sociodemographic information (age, sex, address, occupation, religion, ethnicity.)			X		
	History	Pain history (site of pain, duration of pain, continuous or intermittent pain, aggravating and relieving factors)			X		
		Co-morbidities			X		
		Willingness to participate in a randomized controlled trial.	X				
	Fea sibili ty	Acceptability of random allocation to one of the two groups	X				

For peer review only

For peer review only - http://bmjopen.bmj.com/site/about/guidelines.xhtml

		Acceptability of intervention session (one session in a week with home treatment program throughout the week)	X				
		*Feasibility of blinding the assessor					X
		Eligibility and recruitment rates	X				
		*Acceptability of screening procedures					W
		Understanding possible contamination between the groups					X
		Evaluating the credibility of the intervention			X		X
		Adherence to intervention					X
		Treatment satisfaction					X
		Difficulty in understanding the treatments					X
	Secondary outcomes	PROMIS Pain Interference			X		X
		PROMIS Pain intensity			X		X
		Quality of Life			X		X
		PROMIS Sleep disturbance			X		X
		PROMIS Depression			X		X
		GROC			-		X
		PCS			X		X
		CD-RISC-10			X		X

Note: *Assessed by the therapist providing intervention; all other outcomes are assessed by the blinded outcome assessor.
Abbreviations: T_1, Enrolment time; T_0, Allocation time; T_1, Baseline assessment (before treatment); T_2, During treatment; T_3, One week post-treatment; *PROMIS*, Patient-Reported Outcome Measurement Information System; GROC, Global Rating of Change; *PCS*, Pain Catastrophizing Scale; *CD-RISC-10*, 10-item Connor-Davidson Resilience Scale. *W*= assessment at the end of every week on Fridays.

Participants

Patients with non-specific LBP seeking rehabilitative services at Sahara Physiotherapy Hospital will be invited to participate. Interested patients will be screened by a research assistant (physiotherapist by training) involved in the research.

Inclusion Criteria

For peer review only - http://bmjopen.bmj.com/site/about/guidelines.xhtml

Nonspecific LBP (LBP other than those excluded, see exclusion criteria below) of any duration with pain primarily localized between T12 and gluteal folds, in patients aged 18 years or older, with average pain intensity reported as moderate, severe, or very severe on a PROMIS five-point PROMIS Pain Intensity Short-form Scale[33] over the past week, and who is a Nepalese and is able to understand and speak Nepali fluently will be included in the study.

Exclusion Criteria

Participants with likely specific causes of LBP will be excluded using a triage procedure as suggested by Bardin and colleagues.[34] This includes exclusion of participants having history of prolonged use of corticosteroid, history of malignancy, recent history of fever or chills, history of other diseases associated with compromise in immune system, history of recent spinal surgery or dental procedures, recent history of trauma to spine or a fracture of a spine, history of bladder and bowel dysfunction, history of perineal or saddle anaesthesia, and history of weakness of lower extremity or loss of sensation in lower extremity. Additionally, current pregnancy, and history of diagnosed mental health conditions that would limit adherence to the trial procedures will be excluded.

Sample Size

For peer review only - http://bmjopen.bmj.com/site/about/guidelines.xhtml

For a feasibility study, it is inappropriate to calculate sample size based on desired statistical power to detect a treatment effect,[35] because, the primary aim of the study is to assess if a full trial can or should be conducted. Feasibility outcomes are descriptive in nature; therefore inferential statistics regarding treatment effects will not be computed. To achieve the primary objectives related to feasibility outcomes, the research team estimated that 40 participants would be adequate.[36] Twenty patients will be randomly allocated to each treatment condition.

Participant screening and recruitment

Consecutive participants with non-specific LBP will be invited to participate in this study. The study purpose and procedures will be described to potential participants. This will include information about the benefits and potential harms of the intervention, the time required for the completion of the study, follow-up duration, voluntariness of participation, cost of participation, and the rights to withdraw from the study at any point. A study information sheet will be provided to all potential participants.
If the potential participants are interested in participating, they will be screened for eligibility by a research assistant who is a physiotherapist. If the participants are found eligible, informed consent will be obtained. For those who cannot sign the consent, a witness will sign on their behalf, or the study participant will provide a thumb print on the form for those who cannot write or sign the form as per the ethical guidelines provided by NHRC. We will include uneducated patients who cannot sign an informed

For peer review only - http://bmjopen.bmj.com/site/about/guidelines.xhtml

consent in order to increase the inclusion of uneducated or low education group, given that 31% people in Nepal who are five years old or more cannot read and write.[37] Additionally, exploration of feasibility of pain education in those with no schooling or low educational attainment is important in order to inform clinical practice.

Participants will be informed that they will receive one of the two treatments randomly. It will be highlighted that both of the treatment options are thought to be effective for LBP, and that the goal of the main study is to compare the interventions, however the current study will more specifically evaluate the feasibility of such a study.

Group Allocation, Randomization and Blinding

Random number sequence, in random blocks of 4 and 6, will be generated using www.randomization.com, by a researcher (JHA) who is not involved in recruitment process. Allocation concealment will be performed using opaque, sealed envelopes. The participants will be allocated to one of the two groups by a hospital staff-member who is not the assessor. The two groups will be: (1) Group 1: Pain education group (PEG), and (2) Group 2: Control group (CG).

Intervention

For peer review only - http://bmjopen.bmj.com/site/about/guidelines.xhtml

The TIDieR (Template for Intervention Description and Replication) Checklist was followed when planning the study intervention.[38 39] Manuals of standard operating procedures (MSOPs) will be followed during the delivery of the interventions in both the groups. This will ensure treatment uniformity and fidelity. It is not possible to blind the intervention providers based on the design of the study.

Participants in intervention group (PEG) will receive detailed pain education as described in the next paragraph, and those in the control group (CG) will receive evidence-based physiotherapy treatment. After the completion of the post-treatment assessment at the end of one week, study participants in both the groups will receive the treatment being provided by physiotherapists at Sahara Physiotherapy Hospital. Participants in both the groups will be encouraged not to seek for other medical care for LBP during the 1-week study period, unless they have to. If they do undertake other forms of treatment, they will be requested to report this during the follow-up assessment, and this will be recorded.

Pain education group (PEG)

The Pain Education will be delivered to the PEG only. We will use the Pain Education handouts in Nepali for Nepalese with LBP based on the resources developed by Moseley and Butler, called Explain Pain.[29 40 41] It has evolved in over 15 years[42] and undergone changes and advances.[29 40 41]

For peer review only - http://bmjopen.bmj.com/site/about/guidelines.xhtml

[43,44]Pain education is delivered to provide reassurance, which means removal of fear and concerns about illness.[45] Reassurance is among the core aspects of management of patients with non-specific problems such as LBP.[46] Although there are no gold standard ways to provide reassurance and alleviate fear and concerns about a disease or its consequences, empathy and collaboration are thought to play important role.[47] The Pain Education intervention will be provided by the lead investigator (SS) who is trained in the delivery of this treatment , and has about 10 years of experience in the management of musculoskeletal disorders, including LBP.

In order to develop the Pain Education resources in Nepali, the first step is the development of curriculum for pain education for patients with LBP[29] in Nepal. The curriculum was first outlined in English (by SS) in the similar manner recommended by the developers,[29] and was reviewed and approved by both the developers (see Appendix 1). Based on the curriculum, the Pain Education materials and patient handouts were created (by SS) in Nepali. The clinical cases and patient stories that were compiled are actual stories collected from patients and clinicians in Nepal, but will be anonymously shared to provide reasoning based on contemporary pain biology knowledge.[29] Pictures that are found in Explain Pain resource materials[29 40 41] were adapted in the Nepali version. The Pain Education handout and materials that are produced in Nepali is proof-read and will first be pre-tested in 5 - 10 Nepalese with LBP as

For peer review only - http://bmjopen.bmj.com/site/about/guidelines.xhtml

needed, and corrected, if necessary, before using in the feasibility study participants. The final handouts will then be printed for the use in the current study. This adapted process will ensure that the Nepali pain education materials produced are valid and culturally appropriate. However, for the purpose of the full trial, the difficulty in understanding the treatment will be assessed in the current feasibility study, and any modifications required will be made.

Dosage of the PEG intervention

A single approximately one hour Pain Education will be delivered to the PEG, because evidence indicates that: (1) interventions as brief as five minutes have been shown to have reassuring effects lasting for up to a year,[44] and a single consultation have been found to be as reassuring as the multiple session interventions.[47] We prefer a single session treatment over multiple session treatment, because for many patients in rural Nepal, it is difficult to deliver a multiple session treatment in reality. Thus, we plan to provide a single session delivering key concepts of Explain Pain, which will be reinforced by providing patient handouts to look at and read at home. In fact, a one hour session should be adequate to cover the key Explain Pain concepts by keeping the content simple and jargon-free. Use of a plain language in the health-related education is important to adapt in low health literacy.[48]

Home advice for PEG group

For peer review only - http://bmjopen.bmj.com/site/about/guidelines.xhtml

A printed handout of the pain education material will be provided only to the study participants in the PEG. Participants will be advised to read them, followed by perform physical activity including walking for approximately 30 minutes. Education accompanied by written information has reported to yield the largest effects on fear-component of emotional distress.[49] However, education level of Nepalese is low (65%), we will adapt the written materials to incorporate many more images than text.[37]

For those who cannot read, the family member(s) will be encouraged to read out the materials to them. To complement the written materials, we will also provide an audio recording of the Pain Education session to the patients as an online URL link to Nepali patient education material stored in the cloud, or will be copied to their smart phones or both for those who have the facilities to use them. The Pain Education advice will be directed towards reducing brain's perception movement and exercise as a threat encouraging participants to slowly pace the movement, physical activity, and exercise. This is thought to desensitize the sensitive nervous system, and improve function. A written instruction to perform general exercises and physical activity will be sent to the participants. Participants will be discouraged to use a lumbar corset and rest as coping strategy; whereas physical activity and return to work will be encouraged. A reminder to perform home exercises will be sent to all participants for a total of five days in the week.

For peer review only

For peer review only - http://bmjopen.bmj.com/site/about/guidelines.xhtml

Control group (CG)

The intervention will be provided by the physiotherapists working at the study site. Treatment integrity in the control group will be improved by providing an interactive seminar to all the physiotherapists delivering control group intervention by the lead researcher. The seminar will incorporate evidence-based information, including assessment and management of LBP based on the current recommendations from clinical practice guidelines.[23 50-52] Research articles, and simplified evidence-based summary will be provided to the physiotherapists before the interactive seminar for self-study. At the end of the seminar, a brief multiple-choice quiz will be conducted for the study physiotherapists that will assess evidence-based knowledge and management of LBP of the therapists. Therapists will need to score a minimum of 80% before they deliver treatment to the control group.

Intervention in the control group

The control participants will receive physiotherapy care based on the recent clinical practice guidelines from three different western countries- (1) American College of Physicians, USA (2017),[23] (2) National Institute for Health and Care Excellence, UK (2016),[50 52] and (3) Toward Optimized Practice, Canada (2015).[51] These guidelines are used because they are recent and highly regarded. We did not find any evidence based clinical practice guidelines for the management of LBP in Nepal, or other

For peer review only - http://bmjopen.bmj.com/site/about/guidelines.xhtml

developing countries. The control group interventions were derived by comparing the recommendations made by each of the three guidelines on specific management strategies. The control group treatment components were selected if: (1) two or more guidelines recommended the treatment component , (2) all the authors of the current study agreed that the component should be included , (3) the component was culturally accepted and feasible to deliver in Nepal, (4) the component was determined to be appropriate to deliver at first contact, and (5) the total duration of all of the components would sum up to one hour (approximately) to make the contact hour comparable to the approximately one hour of Pain Education that would be provided to the experimental group. Similarities and differences in the clinical practice guidelines across the countries are common, and are influenced highly from the experience of the expert committee responsible for developing a clinical practice guideline, and local practice trends.[53] For this reason, we chose to adapt the recommendations made in the available guidelines to fit with the expertise of the local physiotherapist, practice trends that are widely accepted in Nepal, and the feasibility of delivering the treatment within the context of the study.

Thus, the control group intervention will contain of: (1) education (advice to remain active, education about prognosis of LBP for acute LBP, and avoid bed rest and braces)[23 51 52] for 10-15 minutes, including time spent to listen to each participant's pain story; (2) back massage[23 52] for about 10 minutes; (3) superficial heat[23 51] for 10-15 minutes; and (4) static cycling or

For peer review only - http://bmjopen.bmj.com/site/about/guidelines.xhtml

(treadmill) walking with the aim to promote physical activity[23 51 52] including any rest period for a total of approximately 20 minutes. Although superficial heat is recommended in the acute/subacute LBP,[23 51] we included this as a common treatment for all types of LBP including chronic LBP, which could be a part of self-management. Any forms of electrotherapy and acupuncture will not be offered to the study participants.[50] The control intervention will strictly exclude the use of pain biology education.

Dosage of intervention for the CG

The control intervention will last for one hour to match the experimental group.

Home advice for the CG

Participants will be advised to self-manage their back pain based on the information provided. Home exercise leaflet with emphasis on the value of exercise to increase strength and endurance, followed by a 30 minutes walking. Advice preceding the exercises will state that exercises are needed to keep you strong, healthy, and pain-free. A written instruction to perform general exercises and physical activity will be sent to the participants. Participants will be discouraged to use a lumbar corset and rest as coping strategy; and return to work and physical activity will be encouraged in the control participants, as they are in the experimental

For peer review only - http://bmjopen.bmj.com/site/about/guidelines.xhtml

group. A reminder to perform home exercises will be sent to all the participants for five days during the week.

Outcome Measures

The details of primary feasibility outcomes are presented in table 2.

For peer review only

For peer review only - http://bmjopen.bmj.com/site/about/guidelines.xhtml

Table 2. How will each primary feasibility objective be assessed?

SN	Objectives	Measures to assess specific objectives	Statistical analysis
1	Willingness to participate in a randomized controlled trial	All the consecutive participants presenting at the physiotherapy department will be approached and invited to participate. The number and rate of participants willing versus not willing to participate will be recorded, as will the reasons for not wanting to participate.	Total number of patients not willing to participate will be summed and reasons for non-participation collated.
2	Feasibility of blinding the assessor	Feasibility of blinding will be assessed by asking the assessor two questions at the end of the follow-up assessment:<br>1.Did you receive any information that indicated to you which group the participant was assigned to?<br>2.How did you receive information about group assignment?<br>Assessor's guess regarding group assignment group will be recorded for each participant. The responses will be coded as correct or incorrect guess.	The frequency and relative rates of "*Yes*" and "*No*" as the answer to the first question will be computed, as will the frequency and relative rates of correct and incorrect guesses. Finally, reasons for guesses will be recorded as verbatim and reported.
3	Eligibility and recruitment rates	The total number of participants screened, found eligible, and recruited will be recorded during the screening and recruitment process. The reasons why the participants were ineligible for study inclusion will be recorded for every participant who did not meet eligibility criteria. Reasons for declining participation will be recorded for every eligible individual who declined participation. Consent rates will also be recorded.	The number of participants screened, who were eligible, who consented to participate, who refused to participate will be computed, reasons for exclusion and refusal will be collated.
4	Acceptability of screening procedures	The lead researcher will interview the physiotherapist(s) screening the potential participants for eligibility by asking the following open-ended question at the end of every week, *"Were there any difficulties or challenges in screening and recruiting the participants the past week?"* The responses will be recorded as "Yes" or "No". If the answer to this question is "Yes", the lead researcher will ask the following open-ended question, "*What were the difficulties and challenges?*". Responses will be written down verbatim. Additionally, the outcome assessor will complete a form every time he/she encounters a difficulty or a challenge during screening. Outcome assessors will also be asked about	The frequency of difficulties or challenges will be counted.<br>The difficulties and challenges will be noted, categorized if possible, and reported. Results will be used to improve the screening procedure in the full trial in future, and therapists' recommendations will be considered.

For peer review only - http://bmjopen.bmj.com/site/about/guidelines.xhtml

		their recommendations for overcoming any challenges that they identify.<br>Time taken to complete the questionnaires will be recorded.	
5	Acceptability of random allocation to a treatment group	Outcome assessor will ask the participants if random allocation to one of the two treatment groups is acceptable to the participants. Responses will be recorded as "Acceptable", "Not acceptable", or "No preference".	The frequencies of each response will be computed separately for each treatment condition.
6	Understanding possible contamination between the groups	Contamination between the groups will be assessed by asking all the study participants the following questions at one week following treatment:<br>1. *"Have you talked to other participants in this study about the intervention they are receiving?"; If yes, further ask, "Was your attitude towards the intervention, or intervention changed after talking to one of the participants in the other group?";*<br>2. *"Are you aware of the intervention that any participants in the other group are receiving in the study?";*<br>3. *Are any of the participants in the other group aware of the type of intervention you are receiving in this study?"*<br>Participants in the control group will be asked the following question: "*Did you read the pain education booklet or watch the video which is one of the components of the intervention group*?"<br>These questions are adapted from a co-twin controlled feasibility trial.[54]	The frequencies of the participants who responded affirmatively to each question will be computed separately for each treatment condition.
7	Credibility and acceptability of the interventions	Treatment credibility will be assessed using five questions adapted from Borkovec and Nau.[55] The questions will be modified to fit "Pain education" and "Control group" as a treatment for patients with LBP, and will be administered to all the participants in both conditions. One, important cultural adaptation for the scoring will be made by changing the numerical scale proposed by Borkovec and Nau to a Verbal Rating Scale, because Numerical Rating Scales have been shown to be unsuitable in Nepalese with musculoskeletal pain; especially in those who are older and have low educational attainment.[56] Responses for each of the five questions will be recorded on a Likert Scale where, 0= *"Not at all",*	Mean of the total scores on the credibility scale will be computed separately for each condition. Between-condition differences in creditability will be evaluated a *t*-test.

For peer review only - http://bmjopen.bmj.com/site/about/guidelines.xhtml

		1= *"A little bit"*, 2= *"Somewhat"*, 3= *"Quite a bit"*, 4= *"Very much."* The questions are: **<u>Questions:</u>** 1. *How logical does the treatment provided seem to you for the management of low back pain?* 2. *How confident would you be that this treatment would be successful in reducing pain?* 3. *How confident would you be in recommending this treatment to a friend or family who also has LBP?* 4. *If you were having low back pain again, would you be willing to undergo such treatment?* 5. *How successful do you feel this treatment would be in reducing pain in other parts of the body, example, knee pain?* All the questions will be asked at baseline and at one week following treatment.	
8	Adherence to the intervention	Adherence to treatment will be assessed by asking the participants in both conditions to maintain a record of daily home treatment that was followed by every participant. The participants will tick mark a box for every day for a total of five days between the two assessment time-points to indicate their adherence to prescribed home exercise program. Additionally, participants will be requested to record any deviation of prescribed home treatment program (example, additional visit to the clinic and use of other interventions). Finally, question regarding non-adherence will be asked.	The total number of treatment adherence days will be summed for each treatment condition separately. Deviation from the treatment protocol will be recorded. Finally, the reasons for non-adherence will also be listed.
9	Satisfaction of treatment	All the participants in both the conditions will be asked to respond to the Patient Global Assessment of Treatment Satisfaction (PGATS) scale at one week following treatment.[57 58] The question asked will be, ''*How satisfied are you overall with the study treatment?*'' Responses to this question are made on a 5-point categorical scale (0 = ''*Very dissatisfied*''; 1 = ''*Dissatisfied*''; 2 = ''*Neutral or no preference*''; 3 = ''*Satisfied*''; 4 = ''*Very satisfied*'').	Mean treatment satisfaction scores will be computed for each treatment condition separately. Between-condition differences will be evaluated using a *t*-test.
10	Difficulty in understanding	All the participants will be asked about the difficulty in understanding the information provided by the physiotherapist.	The differences in the difficulty in understanding the information provided

For peer review only - http://bmjopen.bmj.com/site/about/guidelines.xhtml

	the information provided by the physiotherapist.	The question asked will be, "*How difficult was it for you to understand* ***<u>the information</u>*** *provided by the physiotherapist?*" Responses will be provided on a 5-point Likert Scale, where 1= "*Very easy*", 2= "*Easy*", 3= "*Neither easy nor difficult*", 4= "*Difficult*", 5= "*Very difficult*".	will be compared between the two groups.
11	Difficulty in understanding the instructions for what to do.	All participants will be asked about the difficulty in understanding the instructions provided by the physiotherapist for what to do at home. The question asked will be, "*How difficult was it for you to understand* ***<u>the instructions</u>*** *provided by the physiotherapist for what to do at home?*" using a on a 5-point Likert Scale, where 1= "Very easy", 2= "*Easy*", 3= "*Neither easy nor difficult*", 4= "*Difficult*", 5= "*Very difficult*".	Frequencies and rates of each response category will be computed for each group separately.
	Adverse events	All participants will be asked about any adverse events after treatment. All responses will be recorded verbatim.	The number of adverse events listed will be computed for each treatment condition separately. The responses will be analyzed qualitatively (see text).

For peer review only - http://bmjopen.bmj.com/site/about/guidelines.xhtml

All but one secondary outcome measures have been shown to be reliable and valid in Nepali populations. The single item measure on quality of life is not yet validated at the time of writing the protocol, however, we have included it as a secondary outcome measure because it is a recommended measure by core-outcome sets in clinical trials for LBP.[59] We hypothesize that this measure is comprehensible and will show adequate validity in the Nepalese sample. We will evaluate the validity of the measure before using it in the full clinical trial.

A research assistant will interview all the study participants to make the study procedures consistent, and to allow for the inclusion of participants with little or no education. The interviews will be administered by a physiotherapist, who will be blind to group assignment. Secondary measures include the four Patient-Reported Outcome Measurement Information System (PROMIS) short-form measures assessing Pain Interference,[60] Pain Intensity,[60] Sleep Disturbance,[60] and Depression,[60] as well as the 13-item Pain Catastrophizing Scale (PCS),[61] Connor-Davidson Resilience Scale (CD-RISC),[62] Global Rating of Change (GROC),[63 64] and a single-item quality of life (QOL) scale. All the items in each PROMIS measure will be summed to obtain raw scores for each scale. The raw scores of each measure will then be converted to T-scores, with a mean of 50 and SD of 10 and recorded (www.assessmentcenter.net). Details of the measures with their measurement properties are presented in table 3.

For peer review only - http://bmjopen.bmj.com/site/about/guidelines.xhtml

Table 3. Secondary outcome measures.

Domain	Name of outcome measure	No. of Items	Response Scale	Scoring	Measurement properties of Nepali versions of the scale.
Pain interference	PROMIS Pain Interference short form 6b[60]	6	5 point, Ordinal	Respondents are asked to rate how much pain interfered with their daily life (e.g., enjoyment of life, ability to concentrate, day-to-day activities) in past 7 days on 5-point Likert scales (1 = "*Not at all*", 2 = "*A little bit*", 3 = "*Somewhat*", 4 = "*Quite a bit*", 5 = "*Very much*"). Responses are scored as a T-score that can range from 0-100, with a mean of 50 and SD of 10 in the normative sample.	• Good internal consistency (Cronbach's alpha= 0.85).<br>• Excellent reliability (ICC= 0.80) in a sample of individuals with chronic pain from Nepal.[60]
Pain catastrophizing	Pain Catastrophizing Scale (PCS)[61]	13	5-point, Ordinal	Respondents are asked to indicate the degree or frequency with which they have each catastrophizing response listed when they "… are experiencing pain" on a 5-point Likert scale (0 = "*Not at all*", 1 = "*To a slight degree*", 2 = "*To a moderate degree*", 3 = "*To a great degree*", and 4 = "*All the time*"). The total PCS score can range from 0 to 52, with higher scores representing higher pain catastrophizing.	• Good to excellent internal consistencies: Cronbach's alpha= 0.85- 0.93, and<br>• Excellent test-retest stability (ICC= 0.89- 0.90).<br>• Positive moderate correlations with measures of pain intensity, depression, and anxiety in a sample of individuals with chronic pain from Nepal.[61]
Patient's global rating of change	Global rating of Change (GROC)[58 63 65]	1	1-7, Ordinal	Respondents are asked to rate their "*overall improvement*" their health condition since the study enrolment. Responses can range from 1 to 7 with 4 = "*No change*". Scores greater than 4 indicate greater improvement and scores lower than 4 indicate a perceived worsening in the respondent's health condition.	MIC: 1 point change.[63 64]
Quality of life	Quality-of-Life (QOL) rating	2	5-point, Ordinal	Respondents are asked to rate their general quality of life and general health by responding	Not available during the time of protocol writing the study protocol.

For peer review only

	scale			to the questions:<br>• "*In general, how would you rate your overall quality of life during the past week?*"<br>• *"How would you rate your general health during the past week?"*<br>The response options are 1 = "Very bad", 2= "Bad", 3= "Fair", 4= "Good", and 5= "Very good".[66]<br>Greater score indicate better quality of life.	
Pain intensity	PROMIS Pain Intensity short form 3b[60]	3	5-point, Ordinal	Respondents are asked to rate their current pain, and their worst and average pain intensity in past 7 days on 5-point Likert Scales (1 = "*Had no Pain*", 2 = "*Mild*", 3 = "*Moderate*", 4 = "*Severe*", and 5 = "*Very severe*"). Responses are scored as a T-score that can range from 0-100, with a mean of 50 and SD of 10 in the normative sample.	Good test-retest reliability (Intraclass Correlation Coefficient [ICC]= 0.71) in a sample of individuals with chronic pain from Nepal.[60]
Sleep disturbance	PROMIS Sleep Disturbance short form 8b[60]	7*	5-point, Ordinal	Respondents are asked to rate sleep quality items on a 5-point Likert scale. Responses are scored as a T-score that can range from 0-100, with a mean of 50 and SD of 10 in the normative sample.	Although the Nepali translation of the 8-item Sleep Disturbance short-form showed poor reliability, removal of one item, improved the test-retest stability to excellent. (ICC= 0.78).<br>Good internal consistency of 7-items (Cronbach's alpha = 0.89).[60]
Depression	PROMIS Emotional Distress-Depression-short form 8b[60]	8	5-point, Ordinal	Respondents are asked to indicate the frequency of depressive symptoms in the past 7 days on a 5-point verbal rating scale (1 = "*Never*", 2 = "*Rarely*", 3 = "*Sometimes*", 4 = "*Often*", and 5 = "*Always*"). Responses are scored as a T-score that can range from 0-100, with a mean of 50 and SD of 10 in the normative sample.	• Excellent internal consistency (Cronbach's alpha= 0.93).<br>• Excellent test-retest reliability (ICC= 0.81) in a sample of individuals with chronic pain from Nepal.[60]
Resilience	10-item Connor Davidson Resilience Scale[62]	10	4-point, Ordinal	Respondents are asked to rate each resiliency item on a 5-point Likert scale: 0 = "*Not true at all*", 1 = "*Rarely true*", 2 = "*Sometimes true*", 3 = "*Often true*", and 4 = "*True nearly all the time*".	• Good to excellent internal consistency (Cronbach's alpha= 0.87- 0.90).[62]<br>• Excellent test-retest stability (ICC=0.89).[62]

For peer review only - http://bmjopen.bmj.com/site/about/guidelines.xhtml

				Responses are summed such that total scores range from 0 to 40, with higher scores indicating more resilience.	• Standard error of measurement= 2.42 points.<br>• Minimum detectable change= 6.72 points.<br>• Significant negative and moderate association with the PCS in a sample of individuals with chronic pain from Nepal.[62]
Use of pain medications and other pain treatments	-	-	-	Names, and dosage of pain medication intake will be recorded by the assessor by asking the research participant via interview. Medications will then be categorized into analgesic type (opioids, NSAIDs, sedatives, and anti-seizure medications), as will the number of days each type of medication is taken.<br>Other pain treatments provided to or used by the study participants will also be recorded and classified (e.g., physical therapy, naturopathy). The number of days each treatment was provided to or used by the participants will be recorded.	• No validity data for self-reported analgesic or pain treatment use in Nepali patients available at the time of protocol writing.

*Only seven items out of 8 items will be included, as the measurement properties of the total 8-item was poor, and only after removal of 1 item, the reliability improved.

For peer review only

The risk of adverse events in both the groups are very low. Participants will be asked to choose the amount of home exercises (such as walking) they will perform based on a level that is comfortable. Participants will be asked to change the duration and/or pace of exercises if they feel the initial level is too high.[67] Participants will be asked to record any adverse events that occur, and report these to the researcher. Adverse events in both the groups will be reported and compared between the groups.

Additional measures

Additional questionnaires will be administered to obtain data related to (1) socio-demographic information (age, sex, education level, employment status, income, religion and ethnicity); (2) pain history, including duration of pain, aggravating and relieving factors, other associated comorbidities; and (3) pain location using pain drawings. Other information such as resources required to conduct the trial (e.g., cost), time required to complete the recruitment of desired number of participants will also be recorded.[68] Total duration of home exercises in each group will also be recorded.

Criteria for feasibility

The results of this feasibility trial will indicate if the study as designed is feasible which will inform the decision of progressing to a full trial with the recommendations. The decision will be one of the following: (1) do not proceed to a full trial if any pre-planned changes may not help improve the

For peer review only - http://bmjopen.bmj.com/site/about/guidelines.xhtml

feasibility; (2) modify the protocol further prior to conducting a full trial; (3) continue with the full trial using the same procedures used in the feasibility trial without modifications, however, monitor the study procedures closely; and (4) continue without modifications, as it is in the feasibility trial, close monitoring is not required.[69] The criteria for the feasibility are presented in Table 4.

Table 4. Criteria for feasibility.

	**Full trial is not feasible as designed**		**Proceed to a full trial without modification in the protocol of the feasibility trial**	
**Criteria**	Modify the protocol prior to a full trial if…	Action	Monitor the study procedures closely if…	Close monitoring is not required if…
**Blinding of assessor**	Assessor has a >70% correct guess rate on the group allocation.	Identify ways to improve assessor blinding based on the responses or feedback provided by the assessors.	70-90% blinding is found.	<10% incorrect guess to the group allocation.
**Recruitment rate**	≤1 participant every week	Identify reasons for low participation rates or declining participation. Possible strategies could be changing the study site, increasing the number of study sites, increasing advertising efforts, using incentives for participation.	2-3 participants recruited every week.	≥ 4 participants recruited every week.
**Attrition rate (in both arms)**	>30% total drop-outs at 1 week post-treatment.	Identify possible reasons for drop-outs and ways to improve follow-up participation.	15%-30% total drop-outs at 1 week post-treatment.	<15% total drop-outs at 1 week post-treatment. (www.pedro.org.au)
**Feasibility of outcome assessment**	>20% missing data on the secondary outcome measures.	Reduce the number of outcome measures. Identify and use brief versions of outcomes.	10%-20% missing data on the secondary outcome measures.	<10% missing data on the secondary outcome measures.
**Contamination of**	≥ 15% contamination	Identify reasons for contamination	<15% contamination	0% contamination between the groups.

For peer review only - http://bmjopen.bmj.com/site/about/guidelines.xhtml
For peer review only

**intervention[5] [4]**	between the groups.	and resolve them.	between the groups.	
**Score on the credibility of treatment scale**	Control condition is > .50 SD less credible that EP.	Develop a new control condition for the EP trial, and pilot test to ensure that its credibility rating is < .50 SD different from EP.	-	The credibility scores of the two conditions are w/i .50 SD units of each other.
**Adherence to treatment**	< 50% attend the treatment. session after randomization.	Identify reasons for not attending the treatment session in order to increase attendance in the full trial.	50%-80% of the participants attend the treatment session.	80% or more of the participants attend the treatment sessions.
**Difficulty scale**	If ≥50% participants in the experimental group rate pain education as very difficult, very easy, easy, or neither easy nor difficult.	Make the intervention a little more challenging if more than half participants report 1-3 out of 5 in the difficulty scale, by increasing the depth of the education, by adding more pain biology education content. Whereas, if more than half participants report the intervention as very difficult, reduce the complexity. Re-evaluate complexity in a second cohort of at least five participants to ensure appropriate level of difficulty.	-	≥50% participants in the experimental group rate pain education as a "difficult" intervention (4 out of 5 in the difficulty scale). This is the preferred difficulty level because we want pain education intervention to be difficult enough to challenge participants – but not too easy or very difficult.

For peer review only

Limitation of the Study

As this is a feasibility study, the results of the current study will not provide findings regarding the efficacy of the interventions being tested. The study

For peer review only - http://bmjopen.bmj.com/site/about/guidelines.xhtml

will only evaluate if this research study is viable as a full trial, and inform any recommendations for modification of the protocols for the full trial resulting from the findings of this feasibility trial.

Plan for Supervision and Monitoring

The study will be conducted, and monitored by the lead investigator under the supervision of the co-authors (JHA, MPJ), with assistance from research assistant(s). All the ethical principles as provided by Declaration of Helsinki will be followed by all the members of this research throughout the study. The investigators will not violate any of the rules and ethical principles of Nepal Health Research Council (NHRC). Monitoring for the NHRC will be regulated by the primary investigator and followed by all researchers and research assistants involved in the study.

Plan for data integrity and management

The research data will be collected by a research assistant who will be trained to collect the research data, manage the data by compiling in a file for individual patient. Participant identifiers (including name, address, and contact information) will be removed from the research data, and will be stored separately. Data will be entered in Microsoft Excel. Identification of the groups as intervention and control group will be removed from the excel sheet. Research data will be monitored weekly by scrutinizing entered data. Any errors in entry will be identified (if any) and amended.

For peer review only - http://bmjopen.bmj.com/site/about/guidelines.xhtml

Consent forms will be scanned and stored in password protected computers of the lead researcher and at the University of Otago along with other research data files.

Data analysis plan

Descriptive statistics will be computed to describe the baseline and demographic characteristics of the study participants. As it is a feasibility study, level of significance and hypothesis testing regarding treatment efficacy will not be performed. Effect sizes representing between-group differences in change in the primary and secondary outcomes will be computed, but these effect sizes will not be considered as a criteria for sample size estimation for the full trial, nor as a criteria to proceed to the full trial, because of the inadequate power of the current feasibility study. Treatment effects for the secondary outcome measures will be presented as means, standard deviations, and confidence intervals of the means. Difference between the mean scores of each secondary outcome will be compared with the minimum important change (MIC) values of the outcome measures, if the MIC scores are available. The analysis plans of the primary feasibility objectives are described in table 2.

Patient and Public Involvement

The research question was informed by the clinical observation that many patients from rural Nepal showed significant improvement in their LBP

For peer review only - http://bmjopen.bmj.com/site/about/guidelines.xhtml

outcomes after reassurance and advice to remain physically active. We therefore designed this trial to assess the feasibility of conducting a study to compare the effectiveness of Pain Education and structured evidence-based physiotherapy treatment in Nepalese people with non-specific LBP.

Patients were not involved in the design of the study protocol, but were directly involved in the development of Nepali versions of outcome measures. Patients will also provide feedback and comments in the Nepali Pain Education materials during pre-testing before using it in the feasibility study. Similarly, the development of Nepali Pain Education materials have incorporated real pain-related stories of Nepalese living with pain. The name and identity of all patients were kept confidential. Any information that discloses identity of the patients were excluded in the written Pain Education booklet.

During the initial assessment, all participants will be asked if they would like to know about the results of the study. A plain language summary of the study results will be written both in English and Nepali, which will be published online. The principal investigator of the study (SS) will also post an audio summary of the research results online for those who cannot read. The link of these will be sent to the participants as text messages.

ETHICS AND DISSEMINATION

For peer review only - http://bmjopen.bmj.com/site/about/guidelines.xhtml

The protocol was approved by Nepal Health Research Council (NHRC; registration number: 422/2017) and University of Otago Human Ethics Committee for Health (registration number: H17/157). The results of the study will be presented at national and international conferences, and published in a peer-reviewed journal.

Authors' contributions

SS, JHA, and MPJ conceived the idea of the trial. Trial was designed by SS, JHA, MPJ, and GLM. SS prepared the first draft of the protocol. Successive drafts were edited by JHA, MPJ, and GLM. The final version of the protocol was approved by all the authors.

Funding

None

Competing interests

GLM has received support from Pfizer, AIA Australia, Gallagher Bassett, Kaiser Permanente USA, Port Adelaide Football Club, Arsenal Football Club and the International Olympic Committee. GLM receives royalties for books on pain and rehabilitation, including the text on which the content for the proposed intervention was based. He also receives speaker fees for lectures on pain and rehabilitation. Other authors report no competing interests.

Ethics approval

For peer review only - http://bmjopen.bmj.com/site/about/guidelines.xhtml

The protocol was approved by Nepal Health Research Council (ref number 422/2017) and University of Otago Human Research Committee (Health) (ref number H17/157).

Acknowledgement

The authors would like to thank Associate Prof. David Butler and Tim Cocks from Neuro-Orthopedic Institute (NOI) in Adelaide for their input during the development of curriculum for explaining pain for this study. Dr. Adrian Traeger (University of Sydney) provided valuable suggestions in the earlier version of the protocol. Finally, authors would also like to thank all the patients who agreed to use their pain stories in this Pain Education programme.

For peer review only

For peer review only - http://bmjopen.bmj.com/site/about/guidelines.xhtml

References

1. Balague F, Mannion AF, Pellise F, et al. Non-specific low back pain. *Lancet* 2012;379(9814):482-91. doi: 10.1016/S0140-6736(11)60610-7
2. GBD 2016 Disease and Injury Incidence and Prevalence Collaborators. Global, regional, and national incidence, prevalence, and years lived with disability for 328 diseases and injuries for 195 countries, 1990-2016: a systematic analysis for the Global Burden of Disease Study 2016. *Lancet* 2017;390(10100):1211-59. doi: 10.1016/S0140-6736(17)32154-2
3. Walker BF, Muller R, Grant WD. Low back pain in Australian adults: the economic burden. *Asia Pac J Public Health* 2003;15(2):79-87. doi: 10.1177/101053950301500202
4. Hong J, Reed C, Novick D, et al. Costs associated with treatment of chronic low back pain: an analysis of the UK General Practice Research Database. *Spine (Phila Pa 1976)* 2013;38(1):75-82. doi: 10.1097/BRS.0b013e318276450f
5. Machado GC, Maher CG, Ferreira PH, et al. Trends, Complications, and Costs for Hospital Admission and Surgery for Lumbar Spinal Stenosis. *Spine (Phila Pa 1976)* 2017 doi: 10.1097/BRS.0000000000002207
6. Bhattarai B, Pokhrel PK, Tripathi M, et al. Chronic pain and cost: an epidemiological study in the communities of Sunsari district of

For peer review only - http://bmjopen.bmj.com/site/about/guidelines.xhtml

Nepal. *Nepal Med Coll J* 2007;9(1):6-11. [published Online First: 2007/06/28]

7. Anderson RT. An orthopedic ethnography in rural Nepal. *Med Anthropol* 1984;8(1):46-59. doi: 10.1080/01459740.1984.9965888

8. Bajracharya S, Singh M, Singh GK, et al. Clinico-epidemiological study of spinal injuries in a predominantly rural population of eastern Nepal: A 10 years' analysis. *Indian J Orthop* 2007;41(4):286-9. doi: 10.4103/0019-5413.36988

9. Deyo RA, Nachemson A, Mirza SK. Spinal-fusion surgery - the case for restraint. *N Engl J Med* 2004;350(7):722-6. doi: 10.1056/NEJMsb031771

10. Fritsch CG, Ferreira ML, Maher CG, et al. The clinical course of pain and disability following surgery for spinal stenosis: a systematic review and meta-analysis of cohort studies. *Eur Spine J* 2017;26(2):324-35. doi: 10.1007/s00586-016-4668-0

11. Fernandez M, Ferreira ML, Refshauge KM, et al. Surgery or physical activity in the management of sciatica: a systematic review and meta-analysis. *Eur Spine J* 2016;25(11):3495-512. doi: 10.1007/s00586-015-4148-y

12. Machado GC, Maher CG, Ferreira PH, et al. Non-steroidal anti-inflammatory drugs for spinal pain: a systematic review and meta-analysis. *Ann Rheum Dis* 2017;76(7):1269-78. doi: 10.1136/annrheumdis-2016-210597

13. Enthoven WTM, Roelofs PD, Koes BW. NSAIDs for Chronic Low Back Pain. *JAMA* 2017;317(22):2327-28. doi: 10.1001/jama.2017.4571

For peer review only - http://bmjopen.bmj.com/site/about/guidelines.xhtml

14. Saragiotto BT, Machado GC, Ferreira ML, et al. Paracetamol for low back pain. *Cochrane Database Syst Rev* 2016(6):CD012230. doi: 10.1002/14651858.CD012230

15. Mathieson S, Maher CG, McLachlan AJ, et al. Trial of Pregabalin for Acute and Chronic Sciatica. *N Engl J Med* 2017;376(12):1111-20. doi: 10.1056/NEJMoa1614292

16. Abdel Shaheed C, Maher CG, Williams KA, et al. Efficacy and tolerability of muscle relaxants for low back pain: Systematic review and meta-analysis. *Eur J Pain* 2017;21(2):228-37. doi: 10.1002/ejp.907

17. Abdel Shaheed C, Maher CG, Williams KA, et al. Efficacy, Tolerability, and Dose-Dependent Effects of Opioid Analgesics for Low Back Pain: A Systematic Review and Meta-analysis. *JAMA Intern Med* 2016;176(7):958-68. doi: 10.1001/jamainternmed.2016.1251

18. Saragiotto BT, Maher CG, Yamato TP, et al. Motor Control Exercise for Nonspecific Low Back Pain: A Cochrane Review. *Spine (Phila Pa 1976)* 2016;41(16):1284-95. doi: 10.1097/BRS.0000000000001645

19. Macedo LG, Saragiotto BT, Yamato TP, et al. Motor control exercise for acute non-specific low back pain. *Cochrane Database Syst Rev* 2016;2:CD012085. doi: 10.1002/14651858.CD012085

20. Saragiotto BT, Maher CG, Yamato TP, et al. Motor control exercise for chronic non-specific low-back pain. *Cochrane Database Syst Rev* 2016(1):CD012004. doi: 10.1002/14651858.CD012004

21. Cherkin DC, Anderson ML, Sherman KJ, et al. Two-Year Follow-up of a Randomized Clinical Trial of Mindfulness-Based Stress Reduction

For peer review only

For peer review only - http://bmjopen.bmj.com/site/about/guidelines.xhtml

vs Cognitive Behavioral Therapy or Usual Care for Chronic Low Back Pain. *JAMA* 2017;317(6):642-44. doi: 10.1001/jama.2016.17814

22. Gatchel RJ, Rollings KH. Evidence-informed management of chronic low back pain with cognitive behavioral therapy. *Spine J* 2008;8(1):40-4. doi: 10.1016/j.spinee.2007.10.007 [published Online First: 2008/01/01]

23. Qaseem A, Wilt TJ, McLean RM, et al. Noninvasive Treatments for Acute, Subacute, and Chronic Low Back Pain: A Clinical Practice Guideline From the American College of Physicians. *Ann Intern Med* 2017;166(7):514-30. doi: 10.7326/M16-2367

24. Van Wambeke P, Desomer A, Ailiet L, et al. Low back pain and radicular pain: assessment and management. *KCE Report* 2017;287

25. Almeida M, Saragiotto B, Richards B, et al. Primary care management of non-specific low back pain: key messages from recent clinical guidelines. *Med J Aust* 2018;208(6):272-75.

26. Hurley J, O'Keeffe M, O'Sullivan P, et al. Effect of education on non-specific neck and low back pain: A meta-analysis of randomized controlled trials. *Man Ther* 2016;23:e1-2. doi: 10.1016/j.math.2016.02.009

27. Moseley GL. Evidence for a direct relationship between cognitive and physical change during an education intervention in people with chronic low back pain. *Eur J Pain* 2004;8(1):39-45. doi: 10.1016/S1090-3801(03)00063-6

For peer review only - http://bmjopen.bmj.com/site/about/guidelines.xhtml

28. Moseley GL, Nicholas MK, Hodges PW. A randomized controlled trial of intensive neurophysiology education in chronic low back pain. *Clin J Pain* 2004;20(5):324-30.

29. Moseley G, Butler D. Explain pain supercharged. *Adelaide, Australia: Noigroup publications* 2017

30. Sharma S, Pathak A, Jensen MP. Words that describe chronic musculoskeletal pain: implications for assessing pain quality across cultures. *J Pain Res* 2016;9:1057-66. doi: 10.2147/JPR.S119212 [published Online First: 2016/11/30]

31. Eldridge SM, Chan CL, Campbell MJ, et al. CONSORT 2010 statement: extension to randomised pilot and feasibility trials. *BMJ* 2016;355:i5239. doi: 10.1136/bmj.i5239

32. Chan AW, Tetzlaff JM, Gotzsche PC, et al. SPIRIT 2013 explanation and elaboration: guidance for protocols of clinical trials. *BMJ* 2013;346:e7586. doi: 10.1136/bmj.e7586

33. Chen WH, Revicki DA, Amtmann D, et al. Development and Analysis of PROMIS Pain Intensity Scale. *Quality of Life Research* 2012;20:18-18.

34. Bardin LD, King P, Maher CG. Diagnostic triage for low back pain: a practical approach for primary care. *Med J Aust* 2017;206(6):268-73.

35. Billingham SA, Whitehead AL, Julious SA. An audit of sample sizes for pilot and feasibility trials being undertaken in the United Kingdom registered in the United Kingdom Clinical Research Network

For peer review only - http://bmjopen.bmj.com/site/about/guidelines.xhtml

database. *BMC Med Res Methodol* 2013;13:104. doi: 10.1186/1471-2288-13-104 [published Online First: 2013/08/22]

36. Mengoni SE, Gates B, Parkes G, et al. Wordless intervention for people with epilepsy and learning disabilities (WIELD): a randomised controlled feasibility trial. *BMJ Open* 2016;6(11):e012993. doi: 10.1136/bmjopen-2016-012993
37. Government of Nepal. National Population and Housing Census 2011 (National Report). In: Statistics CBo, ed. Kathmandu: NHPC, 2012.
38. Yamato T, Maher C, Saragiotto B, et al. The TIDieR Checklist Will Benefit the Physical Therapy Profession. *J Orthop Sports Phys Ther* 2016;46(6):402-4. doi: 10.2519/jospt.2016.0108
39. Hoffmann TC, Glasziou PP, Boutron I, et al. Better reporting of interventions: template for intervention description and replication (TIDieR) checklist and guide. *BMJ* 2014;348:g1687. doi: 10.1136/bmj.g1687
40. Butler DS, Moseley GL. Explain Pain 2nd Edn: Noigroup Publications 2013.
41. Moseley GL, Butler DSB. Explain Pain Handbook: Protectometer2015.
42. Moseley GL, Butler DS. Fifteen Years of Explaining Pain: The Past, Present, and Future. *J Pain* 2015;16(9):807-13. doi: 10.1016/j.jpain.2015.05.005 [published Online First: 2015/06/09]
43. Clarke CL, Ryan CG, Martin DJ. Pain neurophysiology education for the management of individuals with chronic low back pain: A systematic review and meta-analysis. *Manual Ther* 2011;16(6):544-49. doi: 10.1016/j.math.2011.05.003

For peer review only - http://bmjopen.bmj.com/site/about/guidelines.xhtml

44. Traeger AC, Hubscher M, Henschke N, et al. Effect of Primary Care-Based Education on Reassurance in Patients With Acute Low Back Pain: Systematic Review and Meta-analysis. *JAMA Intern Med* 2015;175(5):733-43. doi: 10.1001/jamainternmed.2015.0217

45. Linton SJ, McCracken LM, Vlaeyen JW. Reassurance: help or hinder in the treatment of pain. *Pain* 2008;134(1-2):5-8. doi: 10.1016/j.pain.2007.10.002

46. Koes BW, van Tulder M, Lin CW, et al. An updated overview of clinical guidelines for the management of non-specific low back pain in primary care. *Eur Spine J* 2010;19(12):2075-94. doi: 10.1007/s00586-010-1502-y

47. Traeger AC, O'Hagan ET, Cashin A, et al. Reassurance for patients with non-specific conditions - a user's guide. *Braz J Phys Ther* 2017;21(1):1-6. doi: 10.1016/j.bjpt.2016.12.007

48. Stableford S, Mettger W. Plain language: a strategic response to the health literacy challenge. *J Public Health Policy* 2007;28(1):71-93. doi: 10.1057/palgrave.jphp.3200102

49. Traeger AC, Moseley GL, Hubscher M, et al. Pain education to prevent chronic low back pain: a study protocol for a randomised controlled trial. *BMJ Open* 2014;4(6):e005505. doi: 10.1136/bmjopen-2014-005505

50. Bernstein IA, Malik Q, Carville S, et al. Low back pain and sciatica: summary of NICE guidance. *BMJ* 2017;356:i6748. doi: 10.1136/bmj.i6748

For peer review only - http://bmjopen.bmj.com/site/about/guidelines.xhtml

51. Toward Optimized Practice (TOP) Low Back Pain Working Group. Evidence-informed primary care management of low back pain: clinical practice guideline 2015 [Available from: http://www.topalbertadoctors.org/cpgs/885801.

52. National Institute for Health and Care Excellence. Low back pain and sciatica in over 16s: assessment and management 2016 [Available from: https://www.nice.org.uk/guidance/ng59.

53. O'Connell NE, Cook CE, Wand BM, et al. Clinical guidelines for low back pain: A critical review of consensus and inconsistencies across three major guidelines. *Best Practice & Research Clinical Rheumatology* 2017

54. Pinheiro MB, Ho KK, Ferreira ML, et al. Efficacy of a Sleep Quality Intervention in People With Low Back Pain: Protocol for a Feasibility Randomized Co-Twin Controlled Trial. *Twin Res Hum Genet* 2016;19(5):492-501. doi: 10.1017/thg.2016.67

55. Borkovec TD, Nau SD. Credibility of Analogue Therapy Rationales. *J Behav Ther Exp Psy* 1972;3(4):257-60. doi: Doi 10.1016/0005-7916(72)90045-6

56. Pathak A, Sharma S, Jensen MP. The Utility and Validity of Pain Intensity Rating Scales for use in Developing Countries. *Pain Reports* 2018:Accepted for publication.

57. Dworkin RH, Jensen MP, Gould E, et al. Treatment satisfaction in osteoarthritis and chronic low back pain: the role of pain, physical and emotional functioning, sleep, and adverse events. *J Pain* 2011;12(4):416-24. doi: 10.1016/j.jpain.2010.07.009

For peer review only - http://bmjopen.bmj.com/site/about/guidelines.xhtml

58. Dworkin RH, Turk DC, Farrar JT, et al. Core outcome measures for chronic pain clinical trials: IMMPACT recommendations. *Pain* 2005;113(1-2):9-19. doi: 10.1016/j.pain.2004.09.012 [published Online First: 2004/12/29]

59. Chiarotto A, Deyo RA, Terwee CB, et al. Core outcome domains for clinical trials in non-specific low back pain. *Eur Spine J* 2015;24(6):1127-42. doi: 10.1007/s00586-015-3892-3

60. Sharma S, Pathak A, Maharjan R, et al. Psychometric properties of nepali versions of PROMIS short from measures of pain intensity, pain interference, pain behaviour, depressions, and sleep disturbance. *The Journal of Pain* 2018;19(3):S59.

61. Sharma S, Thibault P, Abbott JH, et al. Clinimetric properties of the Nepali version of the Pain Catastrophizing Scale in individuals with chronic pain. *J Pain Res* 2018;11:265-76. doi: 10.2147/JPR.S153061

62. Sharma S, Pathak A, Abbott JH, et al. Measurement properties of the Nepali version of the Connor Davidson resilience scales in individuals with chronic pain. *Health Qual Life Outcomes* 2018;16(1):56. doi: 10.1186/s12955-018-0884-0

63. Sharma S, Palanchoke J, Reed D, et al. Translation, cross-cultural adaptation and psychometric properties of the Nepali versions of numerical pain rating scale and global rating of change. *Health Qual Life Outcomes* 2017;15(1):236. doi: 10.1186/s12955-017-0812-8

64. Sharma S, Palanchoke J, Abbott JH. Cross-Cultural Adaptation and Validation of the Nepali Translation of the Patient-Specific

For peer review only - http://bmjopen.bmj.com/site/about/guidelines.xhtml

Functional Scale. *J Orthop Sports Phys Ther* 2018(0):1-23. doi: 10.2519/jospt.2018.7925

65. Farrar JT, Young JP, Jr., LaMoreaux L, et al. Clinical importance of changes in chronic pain intensity measured on an 11-point numerical pain rating scale. *Pain* 2001;94(2):149-58. doi: S0304-3959(01)00349-9 [pii] [published Online First: 2001/11/03]

66. Crane HM, Van Rompaey SE, Dillingham PW, et al. A single-item measure of health-related quality-of-life for HIV-infected patients in routine clinical care. *AIDS Patient Care STDS* 2006;20(3):161-74. doi: 10.1089/apc.2006.20.161

67. Geraghty AW, Stanford R, Little P, et al. Using an internet intervention to support self-management of low back pain in primary care: protocol for a randomised controlled feasibility trial (SupportBack). *BMJ Open* 2015;5(9):e009524. doi: 10.1136/bmjopen-2015-009524

68. Shanyinde M, Pickering RM, Weatherall M. Questions asked and answered in pilot and feasibility randomized controlled trials. *BMC Med Res Methodol* 2011;11:117. doi: 10.1186/1471-2288-11-117

69. Abbott JH. The distinction between randomized clinical trials (RCTs) and preliminary feasibility and pilot studies: what they are and are not. *J Orthop Sports Phys Ther* 2014;44(8):555-8. doi: 10.2519/jospt.2014.0110

For peer review only - http://bmjopen.bmj.com/site/about/guidelines.xhtml

Explaining Pain in an hour

Curriculum for Nepalese with non-specific low back pain

Learner or patient characteristics

Learners are adult patients who present to a Physiotherapy Department in Kathmandu, Nepal for the management of their low back pain (LBP). This education will be delivered to patients with any duration of LBP. Every participant who understands and can speak Nepali can be the learner for the planned curriculum of Explain Pain. These individuals may have a very low literacy level. Patients who have diagnosed psychiatric illness will be excluded from this intervention.

Deliverer or physiotherapist

A physiotherapist (Saurab Sharma) who has undergone “Explain Pain” course twice (in 2015 and 2017) will deliver Explain Pain to all the participants in the study. The physiotherapist will be supervised by Prof. G. Lorimer Moseley, one of the developers of the concept.

Number of learners or patients

A total of 40 learners will participate in Explain Pain in this study. All interventions will be delivered individually to each participant. No other concurrent interventions will be provided to the patients with Explain Pain, however, patients will be encouraged to perform some exercises at home for one week.

Unique needs of the learners

These learners are from Nepal, many of them may not have any formal education, and education as the core component of treatment may be new to these patients. Thus, this group may be a challenging group to provide Explain Pain intervention. Therefore, the contents are simplified and adapted to their need to match their level of understanding, and culture.

Delivery methods

The study physiotherapist will deliver approximately an hour long session of one-on-one Explain Pain to every study participant. Every participant will be provided with a take away education booklet, which includes details of target concept, pictures and stories to strengthen their education provided by the physiotherapist face-to-face. Participants will read the booklet if they are educated, or the family members can read out for them if they cannot read themselves. All the participants will be provided with an additional audio-visual information on neurophysiology knowledge of pain, if they can operate these at home (or office) to reinforce the learning so that the participants hear this every day before the follow-up for assessment in a week.

Place

For peer review only - http://bmjopen.bmj.com/site/about/guidelines.xhtml

A room at a physiotherapy facility (Sahara Care Hospital) in Kathmandu, Nepal.

Patient consideration

1. Physiotherapist will extract learner's personal goal of treatment. Nepalese generally struggle to bring out their own goals in general, this will be a difficult task.
2. Involvement of family throughout the education session will be encouraged if they accompany the patients. Role of family members will be highlighted in the education program and prognosis of the individuals with LBP.
3. All the participants will be reminded to perform their home-based tasks (reading, listening/watching audio-visual support, and exercising) by a text message or a phone call every day for five days a week.

Aims

The deliverer intends to:

1. Advise patient that educate is an important aspect of treatment of pain.
2. Provide contemporary knowledge about pain biology in relation to low back pain in an individual face-to-face teaching and learning environment.
3. Provide a comprehensive patient education by providing relevant information on graded exposure, pacing, and self-management.
4. Use pictures, metaphors, and relevant stories related to pain to explain details and complexities related to pain.

Objectives

At the end of the session learner will:

1. Have contemporary knowledge of pain biology that is relevant to their low back pain.
2. Understand importance of pain knowledge as a therapy.
3. Use the pain knowledge in changing the danger messages into safety signals, and using exercises to pave the track for recovery.

For peer review only

Explain Pain Curriculum plan

Part 1: Question and answer **[10 – 15 minutes]**

Physiotherapist will first toss two questions.

Question 1- "*Is there anything in particular that you would like to learn about your low back pain, or pain in general?*"

Physiotherapist will answer to any questions that arise.

Question 2- *"Do you know what caused your low back pain? Can you please explain the cause of your low back pain from what you have understood, or what you have been told?"*

Address any misconceptions and acknowledge and appreciate healthy/sound understanding about their pain. Talk about scans if appropriate.

Part 2: Discuss the key concepts of pain biology. **[40 minutes]**

1. Pain is normal and almost everyone gets it in life.
2. Body sends danger signals, and brain decides whether to produce pain.
3. Learning about pain changes pain; and anything associated with it can influence it.
4. Body learns pain and becomes overprotective over time.
5. Additional concept: Pain and tissue damage are poorly related.

[see the table below for the details]

Part 3: Do you want to learn ways to train your system? **[5 – 10 minutes]**

Teach 1 – 2 ways to train the system.

End: Ask if the patient has a cell phone. If he or she has a cell phone, ask if he/she wants to receive a daily reminder to perform home-based tasks, and learn more about pain.

Record the number and send the information daily.

For peer review only - http://bmjopen.bmj.com/site/about/guidelines.xhtml

Explain Pain Curriculum Plan

SN	Target concepts	Time required	Other ways of expressing the target concept	Content	Delivery and resources	Reinforcement/ Experiential learning	Did the patient understand? Assessment
1	Pain is normal and almost everyone gets it in life.	10 minutes	• Everyone has some pain in lifetime so you are not alone.<br>• Pain is normal.<br>• Your LBP is yours, is unique to you and real, and only you can control your pain.	• There is no test for pain or love.<br>• Emotional and physical pain are one.<br>• Pain is always a conscious event.	• Stories<br>• Brief pain epidemiology	• Pain should not be a reason to worry about, and stop you from enjoying life, and fulfilling life goals.	• Ask- so who suffers pain or how many people suffer pain?<br><br>*Answer- almost everyone.*
2	Body sends danger signals, and brain decides whether to produce pain.	10 minutes	• How danger signal travels in the body and how pain is perceived.<br>• Brain is needed to create/ perceive pain.<br>• Human body has danger sensors not pain sensors.<br>• Pain depends on the balance between danger and safety.	• Nociceptive pathway.<br>• Pain is created in the brain.<br>• Ask patient if they had pain when they did not have tissue injury (or use aggravating factors).	• Use a picture/ animation of how danger signals reach brain?<br><br>• Use the earthquake story.	• Have you ever experienced having an injury and no pain?<br>• Have you ever experienced pain when there was no injury?<br>• Use the scale protectometer to describe.	• So what creates pain?<br><br>*Answer: brain!!!*
3	Learning about pain changes pain; and anything associated with it can influence it.	10 minutes	• Knowing about pain can reduce pain.<br>• Education is analgesic.<br>• Retraining your system can reduce sensitization.	• Understanding your pain can reduce your pain.<br>• Wrong understanding can increase your pain.	• Sikha's pain story.	• Hear other's pain stories and analyse how it can be changed.	

For peer review only - http://bmjopen.bmj.com/site/about/guidelines.xhtml

4	Body learns pain and becomes overprotective over time.	10 minutes	• Out of many outputs of the brain, pain is only one protective output.<br>• As pain persists, body systems can be over protective.<br>• Multiple systems protect us from threats, and allow us to learn and heal.<br>• You can train your body systems to be less protective.	• As pain persists, body becomes sensitized and over protective.<br>• This can be changed by training our systems.	• The bending and lifting story.	• Ask the patient what are the other symptoms they get with pain? [Examples are: sweating, no sleep, stress, anxiety, fear, anger etc].	• Ask, do you understand this and think if this is logical?<br>• Reinforce this by saying, this is also scientific.
Optional	Pain and tissue damage are poorly related	5 minutes	• Pain is an unreliable indicator of tissue damage.<br>• Pain and scans do not correlate.	• Tissue stop hurting a long time before they heal.<br>• Recent evidences regarding poor correlation between pain and scan reports.	Bad scans in pain free people.	Reinforce by saying degeneration is like greying of hair, which is normal ageing process. It does not hurt.	

For peer review only

SPIRIT

STANDARD PROTOCOL ITEMS: RECOMMENDATIONS FOR INTERVENTIONAL TRIALS

SPIRIT 2013 Checklist: Recommended items to address in a clinical trial protocol and related documents*

Section/item	Item No	Description	Addressed on page number
**Administrative information**			
Title	1	Descriptive title identifying the study design, population, interventions, and, if applicable, trial acronym	_____1________
Trial registration	2a	Trial identifier and registry name. If not yet registered, name of intended registry	_____3________
	2b	All items from the World Health Organization Trial Registration Data Set	_____________
Protocol version	3	Date and version identifier	_____________
Funding	4	Sources and types of financial, material, and other support	_____36______
Roles and responsibilities	5a	Names, affiliations, and roles of protocol contributors	_____1, 36____
	5b	Name and contact information for the trial sponsor	_____N/A_____
	5c	Role of study sponsor and funders, if any, in study design; collection, management, analysis, and interpretation of data; writing of the report; and the decision to submit the report for publication, including whether they will have ultimate authority over any of these activities	_____N/A_____
	5d	Composition, roles, and responsibilities of the coordinating centre, steering committee, endpoint adjudication committee, data management team, and other individuals or groups overseeing the trial, if applicable (see Item 21a for data monitoring committee)	_____23_____

For peer review only

Introduction

Background and rationale	6a	Description of research question and justification for undertaking the trial, including summary of relevant studies (published and unpublished) examining benefits and harms for each intervention	____5-7_____
	6b	Explanation for choice of comparators	______5______
Objectives	7	Specific objectives or hypotheses	_______7_____
Trial design	8	Description of trial design including type of trial (eg, parallel group, crossover, factorial, single group), allocation ratio, and framework (eg, superiority, equivalence, noninferiority, exploratory)	______6______

Methods: Participants, interventions, and outcomes

Study setting	9	Description of study settings (eg, community clinic, academic hospital) and list of countries where data will be collected. Reference to where list of study sites can be obtained	______8_____
Eligibility criteria	10	Inclusion and exclusion criteria for participants. If applicable, eligibility criteria for study centres and individuals who will perform the interventions (eg, surgeons, psychotherapists)	______9-10____
Interventions	11a	Interventions for each group with sufficient detail to allow replication, including how and when they will be administered	______12-19___
	11b	Criteria for discontinuing or modifying allocated interventions for a given trial participant (eg, drug dose change in response to harms, participant request, or improving/ worsening disease)	______N/A____
	11c	Strategies to improve adherence to intervention protocols, and any procedures for monitoring adherence (eg, drug tablet return, laboratory tests)	______19______
	11d	Relevant concomitant care and interventions that are permitted or prohibited during the trial	______12-19___
Outcomes	12	Primary, secondary, and other outcomes, including the specific measurement variable (eg, systolic blood pressure), analysis metric (eg, change from baseline, final value, time to event), method of aggregation (eg, median, proportion), and time point for each outcome. Explanation of the clinical relevance of chosen efficacy and harm outcomes is strongly recommended	_19-22, 40-46____
Participant timeline	13	Time schedule of enrolment, interventions (including any run-ins and washouts), assessments, and visits for participants. A schematic diagram is highly recommended (see Figure)	____38-39_____

For peer review only - http://bmjopen.bmj.com/site/about/guidelines.xhtml

Sample size	14	Estimated number of participants needed to achieve study objectives and how it was determined, including clinical and statistical assumptions supporting any sample size calculations	_____10______
Recruitment	15	Strategies for achieving adequate participant enrolment to reach target sample size	____10-11_____
**Methods: Assignment of interventions (for controlled trials)**			
Allocation:			
Sequence generation	16a	Method of generating the allocation sequence (eg, computer-generated random numbers), and list of any factors for stratification. To reduce predictability of a random sequence, details of any planned restriction (eg, blocking) should be provided in a separate document that is unavailable to those who enrol participants or assign interventions	_____12______
Allocation concealment mechanism	16b	Mechanism of implementing the allocation sequence (eg, central telephone; sequentially numbered, opaque, sealed envelopes), describing any steps to conceal the sequence until interventions are assigned	_____12______
Implementation	16c	Who will generate the allocation sequence, who will enrol participants, and who will assign participants to interventions	______12______
Blinding (masking)	17a	Who will be blinded after assignment to interventions (eg, trial participants, care providers, outcome assessors, data analysts), and how	______13, 20____
	17b	If blinded, circumstances under which unblinding is permissible, and procedure for revealing a participant's allocated intervention during the trial	_____N/A______
**Methods: Data collection, management, and analysis**			
Data collection methods	18a	Plans for assessment and collection of outcome, baseline, and other trial data, including any related processes to promote data quality (eg, duplicate measurements, training of assessors) and a description of study instruments (eg, questionnaires, laboratory tests) along with their reliability and validity, if known. Reference to where data collection forms can be found, if not in the protocol	19-22, 40-46
	18b	Plans to promote participant retention and complete follow-up, including list of any outcome data to be collected for participants who discontinue or deviate from intervention protocols	____18________

Data management	19	Plans for data entry, coding, security, and storage, including any related processes to promote data quality (eg, double data entry; range checks for data values). Reference to where details of data management procedures can be found, if not in the protocol	____22-23______
Statistical methods	20a	Statistical methods for analysing primary and secondary outcomes. Reference to where other details of the statistical analysis plan can be found, if not in the protocol	_23-24, 40-46__
	20b	Methods for any additional analyses (eg, subgroup and adjusted analyses)	_____N/A_____
	20c	Definition of analysis population relating to protocol non-adherence (eg, as randomised analysis), and any statistical methods to handle missing data (eg, multiple imputation)	_______N/A____
**Methods: Monitoring**			
Data monitoring	21a	Composition of data monitoring committee (DMC); summary of its role and reporting structure; statement of whether it is independent from the sponsor and competing interests; and reference to where further details about its charter can be found, if not in the protocol. Alternatively, an explanation of why a DMC is not needed	_____22-23_____
	21b	Description of any interim analyses and stopping guidelines, including who will have access to these interim results and make the final decision to terminate the trial	____N/A______
Harms	22	Plans for collecting, assessing, reporting, and managing solicited and spontaneously reported adverse events and other unintended effects of trial interventions or trial conduct	_____21________
Auditing	23	Frequency and procedures for auditing trial conduct, if any, and whether the process will be independent from investigators and the sponsor	_____N/A______
**Ethics and dissemination**			
Research ethics approval	24	Plans for seeking research ethics committee/institutional review board (REC/IRB) approval	_____25_____
Protocol amendments	25	Plans for communicating important protocol modifications (eg, changes to eligibility criteria, outcomes, analyses) to relevant parties (eg, investigators, REC/IRBs, trial participants, trial registries, journals, regulators)	_______N/A_____

Consent or assent	26a	Who will obtain informed consent or assent from potential trial participants or authorised surrogates, and how (see Item 32)	______11______
	26b	Additional consent provisions for collection and use of participant data and biological specimens in ancillary studies, if applicable	______N/A_____
Confidentiality	27	How personal information about potential and enrolled participants will be collected, shared, and maintained in order to protect confidentiality before, during, and after the trial	______23-25____
Declaration of interests	28	Financial and other competing interests for principal investigators for the overall trial and each study site	______36_____
Access to data	29	Statement of who will have access to the final trial dataset, and disclosure of contractual agreements that limit such access for investigators	______N/A____
Ancillary and post-trial care	30	Provisions, if any, for ancillary and post-trial care, and for compensation to those who suffer harm from trial participation	_____N/A______
Dissemination policy	31a	Plans for investigators and sponsor to communicate trial results to participants, healthcare professionals, the public, and other relevant groups (eg, via publication, reporting in results databases, or other data sharing arrangements), including any publication restrictions	______3, 25_____
	31b	Authorship eligibility guidelines and any intended use of professional writers	______36______
	31c	Plans, if any, for granting public access to the full protocol, participant-level dataset, and statistical code	_______N/A____
**Appendices**			
Informed consent materials	32	Model consent form and other related documentation given to participants and authorised surrogates	_____No______
Biological specimens	33	Plans for collection, laboratory evaluation, and storage of biological specimens for genetic or molecular analysis in the current trial and for future use in ancillary studies, if applicable	_____N/A_____

*It is strongly recommended that this checklist be read in conjunction with the SPIRIT 2013 Explanation & Elaboration for important clarification on the items. Amendments to the protocol should be tracked and dated. The SPIRIT checklist is copyrighted by the SPIRIT Group under the Creative Commons "Attribution-NonCommercial-NoDerivs 3.0 Unported" license.
